# Real‐World Effectiveness and Safety of *Saccharomyces boulardii* CNCM I‐745 as Adjunct Therapy for *Helicobacter pylori* Eradication: Data From the European Registry on *H. pylori* Management (Hp‐EuReg)

**DOI:** 10.1111/hel.70119

**Published:** 2026-04-01

**Authors:** Olga P. Nyssen, Laimas Jonaitis, Ángeles Pérez‐Aísa, Bojan Tepes, Umud Mahmudov, Irina Voynovan, Samuel J. Martínez‐Domínguez, Luis Bujanda, Alfredo J. Lucendo, Ludmila Vologzanina, Ana Garre, Frode Lerang, Sayar R. Abdulkhakov, Matteo Pavoni, Maja Denkovski, Emin Mammadov, Mārcis Leja, Javier Tejedor‐Tejada, Jose M. Huguet, Galyna Fadieienko, Ilchishina Tatiana, Manuel Pabón‐Carrasco, Aiman S. Sarsenbaeva, Oleg Zaytsev, Gülüstan Babayeva, Jesús Barrio, Miguel Areia, Monica Perona, Óscar Núñez, Antonietta G. Gravina, Sergey Alekseenko, Blas José Gómez Rodríguez, Quliyev Fərid Vidadi Oğlu, Inmaculada Ortiz‐Polo, Antonio Moreno Loro, György Miklós Buzás, Boris D. Starostin, Juozas Kupcinskas, Dmitry S. Bordin, Antonio Gasbarrini, Oleksiy Gridnyev, Ricardo Marcos‐Pinto, Manuel Jiménez‐Moreno, Mónica Sánchez Alonso, Virginia Flores, Irene Arteagoitia, Anna Cano‐Català, Pablo Parra, Leticia Moreira, Javier P. Gisbert, the Hp‐EuReg investigators, Boris D. Starostin, Sotirios D. Georgopoulos, Doron Boltin, Manuel Domínguez Cajal, Galina N. Tarasova, Fernando Bermejo, Ludmila V. Morkovkina, Cem Simsek, Perminder S. Phull, María Soledad Marcos, Giuseppe Losurdo, Judith Gomez‐Camarero, Emin Verdiyev, Thomas J. Butler, Sinead M. Smith, Pedro Almela, Antonio Mestrovic, Natalya N. Dekhnich, Pilar Mata‐Romero, Daniel Martin‐Holgado, Marinko Marušić, Ian L. P. Beales, Sabir Sagdati, Dmitry N. Andreev, Igor G. Bakulin, Ivan Nagorni, Alla Kononova, Vladimir Milivojevic, Noelia Alcaide, Benito Velayos, Luis Fernández‐Salazar, Georges Kamtoh, Eduardo Iyo, Pablo M. Wolfe García, Natalia V. Bakulina, Ramón Pajares Villarroya, Miguel Fernández‐Bermejo, Jurij Bednarik, Debora Compare, Sabina Hrubá, Marina F. Osipenko, Dan L. Dumitrascu, Alisan Kahraman, Emilija Nikolovska Trpchevska, Montserrat Planella, Consuelo Ramírez, Victor A. Kamburov, Teresa Angueira, Natalia V. Baryshnikova, Ana Beatriz Pozo Blanco, Pedro Delgado, Maria A. Livzan, Melanija Razov Radas, Natalya V. Bakanova, Eva Barreiro Alonso, Michael Doulberis, Rosario Antón Ausejo, Piotr Eder, Natasa Brglez Jurecic, Wojciech Marlicz, Sheyla Montori Pina, Antonio Cuadrado, Jan Kral, Palencia Palencia, Christos Liatsos, Olga A. Kolokolnikova, Manon C. W. Spaander, Piotr Szredzki, María Badía Martínez, Stergios N. Kouvaras, Mila Kovacheva‐Slavova, Regina I. Khlynova, Benito Hermida Pérez, Petra Čavajdová, Sergio Gil Rojas, Luis Hernández, Ekaterina Y. Plotnikova, Xhensila Pemaj, Deirdre McNamara, Guillem Soy, Ioannis Linas, Riccardo Vasapolli, Marko Nikolic, Andreas Blesl, Tamara Matysiak‐Budnik, Diego Burgos‐Santamaría, Rashad A. Hasanov, Lumir Kunovsky, Carlos Maroto‐Martín, Pilar Bernal Checa, Paola Chaudarcas, Pilar Pazo Mejide, Giulia Fiorini, Ramiro Carreño Macián, Rosa Rosania, Anna‐Maria Tiefenthaller, Teresa Valdés‐Lacasa, Amir Mari, Anna L. Pakhomova, Jan Bornschein, Suzanne Cauchi, Jesus M. Gonzalez‐Santiago, Petra Koňaříková, Isabel Pérez‐Martínez, David Přidal, Jorge Yebra, Miguel Suárez Matías, Natalie Friedova, Diego Ledro Cano, Mirjana Kalauz, Jose Xavier Segarra Ortega, Adam Vasura, Senador Moran Sanchez, Petr Bauer, Katarina Jankovic, Sara Hoxha, Marjan Stankovic, Hagai Schweistein, Alma Keco‐Huerga, Ismar Hasukić, Thomas Balanis, Jakub Langner, Patrick Dinkhauser, Patricia Sanz‐Segura, Melek Balamir, Theodore Rokkas, Mikel Ganuza, Milica Bjelakovic, Antonia Perelló, Marta Pascual‐Mato, Alexander Link, Marino Venerito, Jan Krivinka, Cristina Maria Sabo, Lyudmila Boyanova, Eduardo Albéniz, Skerdi Prifti, Pierre Ellul, Katja Repitsch, Ramazan Erdem Er, Skender Telaku

**Affiliations:** ^1^ Department of Gastroenterology Hospital Universitario de La Princesa, Instituto de Investigación Sanitaria Princesa (IIS‐Princesa), Universidad Autónoma de Madrid (UAM), and Centro de Investigación Biomédica en Red de Enfermedades Hepáticas y Digestivas (CIBERehd) Madrid Spain; ^2^ Department of Gastroenterology Lithuanian University of Health Sciences Kaunas Lithuania; ^3^ Department of Gastroenterology, Hospital Universitario Costa del Sol, RICAPPS ‐ Red de Investigación en Cronicidad, Atención Primaria y Prevención y Promoción de la Salud Marbella Spain; ^4^ Department of Gastroenterology DC Rogaska Slovenia; ^5^ Memorial Hospital Baku Azerbaijan; ^6^ A.S. Loginov Moscow Clinical Scientific Center Moscow Russia; ^7^ Department of Gastroenterology Hospital Clínico Universitario Lozano Blesa, Instituto de Investigación Sanitaria de Aragón (IIS Aragón), Centro de Investigación Biomédica en Red de Enfermedades Hepáticas y Digestivas (CIBERehd) Zaragoza Aragon Spain; ^8^ Department of Gastroenterology Biodonostia Health Research Institute, Department of Medicine, Universidad del País Vasco (UPV/EHU), Centro de Investigación Biomédica en Red de Enfermedades Hepáticas y Digestivas (CIBERehd) San Sebastián Spain; ^9^ Department of Gastroenterology Hospital General de Tomelloso, Instituto de Investigación Sanitaria Princesa (IIS‐Princesa), Centro de Investigación Biomédica en Red de Enfermedades Hepáticas y Digestivas (CIBERehd), Instituto de Investigación Sanitaria de Castilla‐La Mancha (IDISCAM) Tomelloso Spain; ^10^ Gastrocentr Perm Russia; ^11^ Østfold Hospital Trust Grålum Norway; ^12^ Kazan (Volga Region) Federal University, Kazan State Medical University Kazan Russia; ^13^ Department of Medical and Surgical Sciences, Sant'orsola‐Malpighi University Hospital Bologna Italy; ^14^ Interni Oddelek, Diagnostic Centre Slovenia; ^15^ Department of Therapy Azerbaijan State Advanced Training Institute for Doctors named after A. Aliyev, and Memorial Hospital Baku Azerbaijan; ^16^ Department of Gastroenterology Digestive Diseases Centre, Institute of Clinical and Preventive Medicine, University of Latvia Riga Latvia; ^17^ Department of Gastroenterology, Hospital Universitario de Cabueñes Gijón Asturias Spain; ^18^ Department of Gastroenterology, Hospital General Universitario de Valencia Valencia Spain; ^19^ Departments the Division for the Study of the Digestive Diseases and Its Comorbidity With Noncommunicable Diseases Government Institution L.T. Malaya Therapy National Institute of NAMS of Ukraine Kharkiv Ukraine; ^20^ Department of Gastroenterology SM‐Clinic Saint‐Petersburg Russia; ^21^ Department of Gastroenterology Hospital Universitario Virgen de Valme Sevilla Spain; ^22^ Department of Gastroenterology Chelyabinsk Regional Clinical Hospital Chelyabinsk Russia; ^23^ First Clinical Medical Centre Kovrov Russia; ^24^ Department of Gastroenterology Hospital Universitario Río Hortega, Gerencia Regional de Salud de Castilla y León (SACYL) Valladolid Spain; ^25^ Department of Gastroenterology Portuguese Oncology Institute of Coimbra, Faculty of Medicine of the University of Porto (FMUP), RISE@CI‐IPO (Health Research Network), Portuguese Oncology Institute of Porto (IPO Porto) Coimbra Portugal; ^26^ Department of Gastroenterology Hospital Quirón Marbella Marbella Spain; ^27^ Department of Gastroenterology Hospital Universitario La Moraleja, Faculty of Medicine, Universidad Francisco de Vitoria Madrid Spain; ^28^ Department of Hepatogastroenterology University of Study of Campania 'L.Vanvitelli' Naples Italy; ^29^ Department of Hospital Therapy Far Eastern State Medical University Khabarovsk Russia; ^30^ Department of Gastroenterology Hospital Universitario Virgen Macarena Sevilla Spain; ^31^ National Oncology Centre Baku Azerbaijan; ^32^ Department of Gastroenterology Hospital Universitario y Politécnico la Fe Valencia Spain; ^33^ Department of Gastroenterology Hospital Universitario Virgen del Rocío Sevilla Spain; ^34^ Department of Gastroenterology Ferencváros Health Center Budapest Hungary; ^35^ Saint‐Petersburg State Budgetary Institution Healthcare City Policlinic 38 Saint‐Petersburg Russia; ^36^ Department of Pancreatic Biliary and Upper Digestive Tract Disorders, A. S. Loginov Moscow Clinical Scientific Center, Department of Outpatient Therapy and Family Medicine, Tver State Medical University, Department of Propaedeutic of Internal Diseases and Gastroenterology, Russian University of Medicine Moscow Russia; ^37^ Department of Internal Medicine and Gastroenterology Fondazione Policlinico Universitario Agostino Gemelli IRCCS Rome Italy; ^38^ Department of Gastroenterology Centro Hospitalar Do Porto, Instituto De Ciências Biomédicas de Abel Salazar, Universidade do Porto, Center for Research in Health Technologies and Information Systems (CINTESIS) Porto Portugal; ^39^ Department of Gastroenterology Hospital Universitario de Burgos Burgos Spain; ^40^ Department of Gastroenterology Hospital Universitario Santa Bárbara Puertollano Spain; ^41^ Department of Gastroenterology Hospital General Universitario Gregorio Marañón Madrid Spain; ^42^ Department of Gastroenterology Hospital Universitario de Cruces Spain; ^43^ Gastrointestinal Oncology, Endoscopy and Surgery (GOES) Research Group, Althaia Xarxa Assistencial Universitària de Manresa, Institut de Recerca i Innovació en Ciències de la Vida i de la Salut de la Catalunya Central (IRIS‐CC) Manresa Spain; ^44^ Department of Gastroenterology Hospital Clínic de Barcelona, Centro de Investigación Biomédica en Red en Enfermedades Hepáticas y Digestivas (CIBERehd), Institut D'investigacions Biomèdiques August pi i Sunyer (IDIBAPS), University of Barcelona Barcelona Spain

**Keywords:** effectiveness, eradication therapy, *Helicobacter pylori*, hp‐EuReg, probiotics, *saccharomyces boulardii*, safety

## Abstract

**Background:**

*Saccharomyces boulardii* CNCM I‐745 (Sb) is one of the most widely used probiotics in clinical practice. The aim of this study was to assess the impact of adding Sb to *Helicobacter pylori* eradication therapy on treatment outcomes (effectiveness and safety) in routine European gastroenterology clinical practice.

**Materials and Methods:**

Treatment‐naive cases from the European registry on *H. pylori* management (Hp‐EuReg) collected from 2013 to 2023 at AEG‐REDCap were analyzed. Effectiveness was assessed by modified intention‐to‐treat, by treatment and geographic regions. Multivariate analysis identified factors independently associated with eradication success and adverse event incidence.

**Results:**

Among 69,922 cases, probiotics were used in 16,528 (24%) treatments, of which 4404 (27%) included Sb. Sb use was significantly associated with an increase in effectiveness (OR = 2.32; 95% confidence interval, 1.38–4.03; *p* < 0.01) only when prescribed with concomitant therapy encompassing a proton pump inhibitor plus clarithromycin‐amoxicillin‐metronidazole. In addition, a significant reduction in the overall incidence of at least one adverse event was observed in the Sb group (OR = 0.803; 0.66–0.97; *p* < 0.05). Specifically, diarrhea, nausea, dyspepsia, abdominal pain, asthenia, anorexia, heartburn, and dysgeusia occurred significantly less frequently with Sb. Treatment compliance was high in both groups (with and without Sb).

**Conclusions:**

In Europe, the addition of Sb to first‐line regimens in clinical practice was associated with higher effectiveness when combined with concomitant therapy with clarithromycin‐amoxicillin‐metronidazole and with fewer overall adverse events, supporting its role as a beneficial adjunct in *H. pylori* eradication therapy.

**Registration Number:**

ClinicalTrials.gov identifier: NCT02328131

## 1 Introduction

*Helicobacter pylori* ( *H. pylori* ) infects over half of the global population and is the most important risk factor for peptic ulcer and gastric cancer development, making eradication a key public health goal.

The currently recommended first‐line empirical therapies include the administration of a proton pump inhibitor (PPI) together with at least two antibiotics [1], but increasing antibiotic resistance [2, 3], and treatment‐related adverse events (AEs) limit effectiveness [4].

Adjunctive strategies, such as the use of statins [5] or probiotics [2, 6, 7, 8], including *Saccharomyces boulardii* CNCM I‐745 (Sb), have shown promise in reducing gastrointestinal side effects and improving eradication rates [9, 10, 11, 12, 13]. The Maastricht VI/Florence Consensus Report [1] recommends probiotics, including *Saccharomyces boulardii*, to reduce gastrointestinal symptoms during eradication therapy through microbiome regulations [14] and lowering therapy‐related AEs [12, 13, 15]. While meta‐analyses support Sb's benefits [12], real‐world European data are limited. The present study uses the multinational European Registry on *H. pylori* Management (Hp‐EuReg) database to evaluate Sb's effectiveness and safety in routine clinical practice.

## 2 Materials and Methods

### 2.1 European Registry on *H. pylori* Management (Hp‐EuReg)

Hp‐EuReg (www.hpeureg.com) is a multicenter, prospective, non‐interventional registry promoted by the European Helicobacter and Microbiota Study Group, enrolling *H. pylori* ‐infected adult patients since 2013 [16]. The study adheres to the Declaration of Helsinki [17] and was approved by the Ethics Committee of Hospital Universitario de La Princesa (NCT02328131). Data were recorded at e‐CRF AEG‐REDCap [18, 19]. Further details on variables and country selection are detailed in the protocol [16].

### 2.2 Data Management, Selection Criteria and Statistical Analysis

Data were cleaned and quality checked, and only adults receiving first‐line empirical therapy were included; treatments were categorized by probiotic use with Sb (*S. boulardii* CNCM I‐745) as a single strain adjunct identified, while non‐probiotic users (i.e., patients not receiving any probiotic) served as controls. All probiotic prescriptions were independently reviewed and coded by strain.

Clinical and geographic variables were categorized: European regions (Table S1), with ≥ 100 treatments and where Sb was marketed; PPIs were standardized to omeprazole equivalents and classified by dose (low, standard, and high [20, 21]); and treatment duration grouped into 7, 10, or 14 days.

Effectiveness was assessed using a modified ITT (mITT), and per protocol (PP) analyses, with the current study focusing on mITT as the measure closest to real‐world practice. mITT included patients with completed follow‐up and a confirmatory test regardless of compliance; PP included those with completed follow‐up and ≥ 90% treatment adherence.

Safety was assessed by the incidence of at least one AE occurring during eradication therapy. Adherence was defined as taking ≥ 90% of prescribed drugs.

Univariate sub‐analyses assessed effectiveness and safety using Chi^2^ test or Fisher's exact tests followed by backward stepwise logistic regression to identify independent predictors of mITT eradication and AEs, including age, sex (female [reference] vs. male), indication (non‐ulcer [reference] vs. ulcer), duration of treatment (7 days [reference] vs. 10 or 14 days), PPI dosage (low [reference] vs. standard or high dose), compliance (No [reference] vs. Yes), geographic region, Sb use (No [reference]; Yes), and first‐line regimens (six categories): (1) triple therapy with clarithromycin (C) and either amoxicillin (A) or metronidazole (M) [reference]; (2) triple therapy with A and levofloxacin (L); (3) non‐bismuth quadruple concomitant therapy with C, A and either M or tinidazole (T), (4) bismuth‐based quadruple therapy encompassing either the classical format with M, tetracycline (Tc) all given separately or all together in a three‐in‐one single capsule (Pylera); (5) bismuth‐based quadruple concomitant therapy with C and A; and (6) ‘other regimens’ (≤ 10% of the regimens overall). Results were reported as odds ratios (OR) and 95% confidence intervals (CIs) (*p* < 0.05).

## 3 Results

### 3.1 Population Characteristics and Treatment Patterns

**FIGURE 1:**
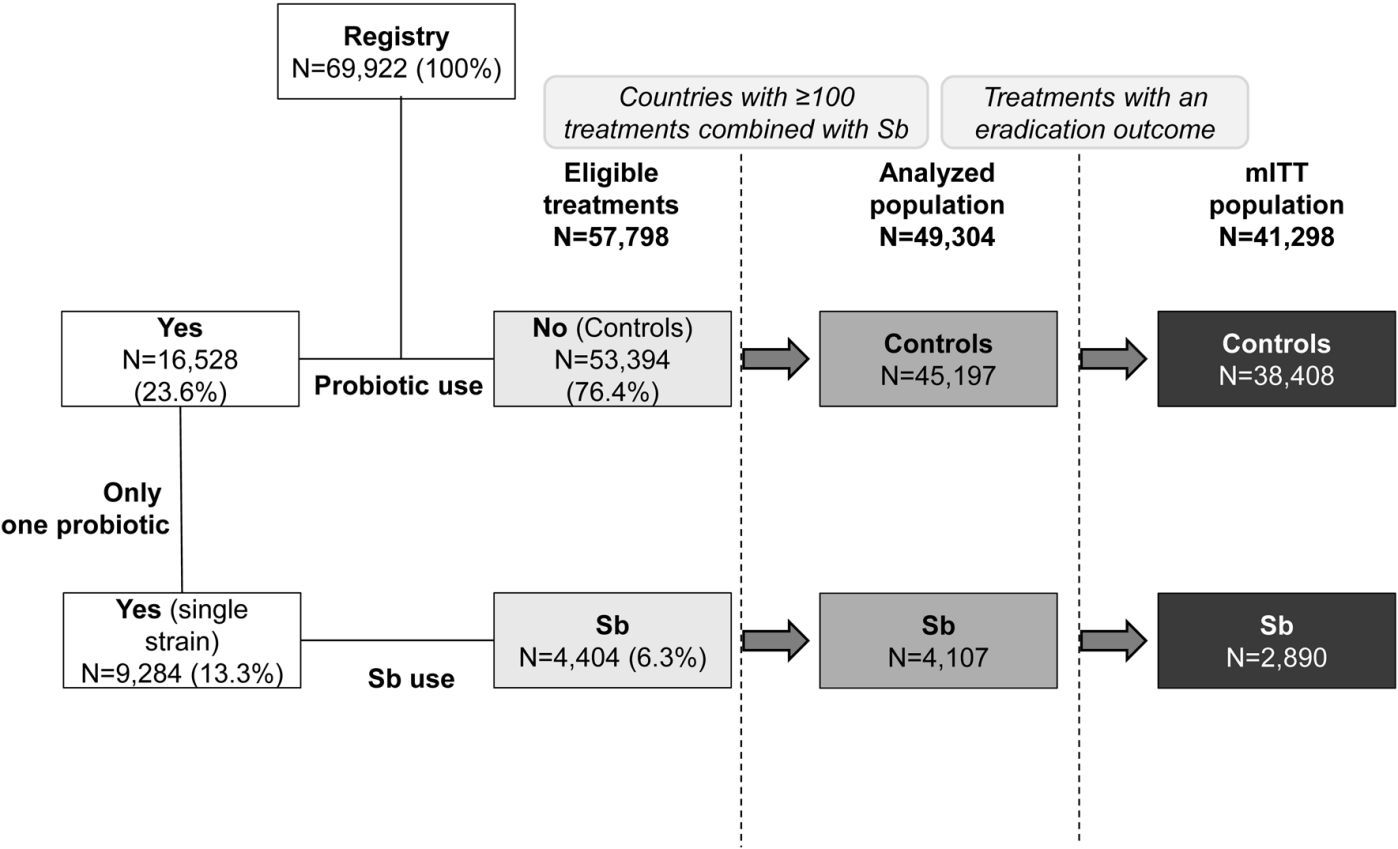
Study flow chart. mITT: Modified Intention‐To‐Treat (population); N, number of treatments; Sb, *saccharomyces boulardii* CNCM I‐745 (single‐strain).

From May 2013 to December 2023, the Hp‐EuReg recorded 69,922 first‐line empirical eradication treatments. Of these, 53,394 were administered without probiotics (control group) and 16,528 (31%) with at least one (Figure 1); and 4404 treatments used Sb (6% of all treatments; 27% of probiotic combined treatment). Among the 57,798 eligible treatments (Sb and control), 41,298 were included in the mITT population (Figure 1).

Patients were mostly female (60%), Caucasian (88%), and aged between 31 and 70 years (79%) (Table 1). Treatments were mainly for non‐investigated dyspepsia or dyspepsia with normal endoscopy (70%); with 9% and 7% for duodenal and gastric ulcers, respectively. One‐third (34%) of patients took concomitant medication including PPIs (37%) and statins (27%); with < 5% on daily non‐steroidal anti‐inflammatory drugs (NSAIDs; 3%). Dyspepsia was the most common symptom (76%). *H. pylori* infection was diagnosed using histology (38%), rapid urease test (38%), and 13C‐urea breath test (26%).

**TABLE 1:** Baseline demographic and clinical characteristics by group.

Characteristics		Total (*N* = 57,798)		Control group (*N* = 53,394)		Sb group (*N* = 4404)		*p*
Gender	*N*	57,709		53,308		4401		0.329^a^
	Female	34,646	60.0%	31,974	60.0%	2673	60.7%	
	Male	23,063	40.0%	21,335	40.0%	1728	39.3%	
Ethnic group	*N*	57,720		53,323		4397		0.000^b^
	Caucasian	50,673	87.8%	47,174	88.5%	3499	79.6%	
	Black	378	0.7%	344	0.6%	34	0.8%	
	Asian	618	1.1%	541	1.0%	77	1.8%	
	Other	4949	8.6%	4243	8.0%	706	16.1%	
	Unknown	1102	1.9%	1021	1.9%	81	1.8%	
Age (years)	*N*	57,589		53,193		4396		0.15^b^
	18–30	6395	11.1%	5909	11.1%	486	11.1%	
	31–50	22,054	38.3%	20,464	38.5%	1590	36.2%	
	51–70	23,612	41.0%	21,743	40.9%	1869	42.5%	
	71+	5528	9.6%	5077	9.5%	451	10.3%	
Drug allergies	*N*	57,724		4402		53,322		0.002^a^
Ooverall	Yes	2401	4.2%	2180	4.1%	221	5.0%	
	No	55,323	95.8%	51,142	95.9%	4181	95.0%	
Indication	*N*	57,708		53,306		4402		0.000^b^
	Dyspepsia (normal endoscopy)	28,411	49.2%	26,761	50.2%	1650	37.5%	
	Dyspepsia (non‐investigated)	12,159	21.1%	11,008	20.7%	1151	26.1%	
	Duodenal ulcer	5268	9.1%	4861	9.1%	407	9.2%	
	Gastric ulcer	4052	7.0%	3646	6.8%	406	9.2%	
	Preneoplastic lesions	1669	2.9%	1475	2.8%	194	4.4%	
Concomitant	*N*	57,677		53,276		4401		0.740^a^
treatments	Yes	19,662	34.1%	18,172	34.1%	1490	33.9%	
PPIs	*N*	19,599		18,112		1487		0.000^b^
	On demand	4286	21.9%	4050	22.4%	236	15.9%	
	Daily	7345	37.5%	6820	37.7%	525	35.3%	
	No	7638	39.0%	6918	38.2%	720	48.4%	
Aspirin	*N*	19,592		18,107		1485		0.000^b^
	On demand	332	1.7%	322	1.8%	10	0.7%	
	Daily	2618	13.4%	2368	13.1%	250	16.8%	
	No	16,118	82.3%	14,898	82.3%	1220	82.2%	
NSAIDs	*N*	19,594		18,112		1482		0.000^b^
	On demand	3893	19.9%	3728	20.6%	165	11.1%	
	Daily	672	3.4%	643	3.6%	29	2.0%	
	No	14,502	74.0%	13,228	73.0%	1274	86.0%	
Statins	*N*	19,595		18,111		1484		0.000^b^
	On demand	137	0.7%	133	0.7%	4	0.3%	
	Daily	5306	27.1%	4922	27.2%	384	25.9%	
	No	13,624	69.5%	12,531	69.2%	1093	73.7%	
Gastrointestinal	*N*	57,747		53,343		4404		
symptoms	No	4916	8.5%	4259	8.0%	657	14.9%	0.000^a^
	Heartburn	14,899	25.8%	13,991	26.2%	908	20.6%	0.000^a^
	Dyspepsia	44,054	76.3%	41,012	76.9%	3042	69.1%	0.000^a^
	Other	7776	13.5%	7192	13.3%	584	13.5%	0.695^a^
Diagnosis	*N*	57,747		53,343		4404		
	13C‐UBT	15,045	26.1%	14,320	26.8%	725	16.5%	0.000^a^
	Serology	4227	7.3%	3706	6.9%	521	11.8%	0.000^a^
	Histology	22,197	38.4%	20,495	38.4%	1702	38.6%	0.772^a^
	Rapid urease test	22,177	38.4%	20,606	38.6%	1571	35.7%	0.000^a^
	Culture	5407	9.4%	5217	9.8%	190	4.3%	0.000^a^
Antibiotic	*N*	57,747		53,343		4404		
resistance	No resistance	2051	3.6%	2008	3.8%	43	1.0%	0.000^a^
	Clarithromycin	1937	3.6%	1914	3.6%	23	0.5%	0.000^a^
	Nitroimidazole	1814	3.1%	1793	3.4%	21	0.5%	0.000^a^
	Quinolone	1283	2.2%	1264	2.4%	19	0.4%	0.000^a^
	Amoxicillin	50	0.1%	49	0.1%	1	0.0%	0.181^a^
	Tetracycline	26	0.0%	26	0.0%	0	0.0%	0.259^a^
	Not performed	927	1.7%	907	1.7%	20	0.5%	0.000^a^
Geographical	*N*	57,747		53,343		4404		0.000^b^
distribution	Eastern	12,206	21.1%	10,407	19.5%	1799	40.8%	
European	Central–Eastern	15,062	26.1%	14,662	27.5%	400	9.1%	
regions	South‐Western	21,745	37.7%	20,656	38.7%	1089	24.7%	
	Central‐Western	5737	9.9%	4777	9.0%	960	21.8%	
	Northern	2997	5.2%	2841	5.3%	156	3.5%	
Geographical	*N*	57,747		53,343		4404		0.000^b^
distribution	Russia	9450	16.4%	7750	14.5%	1700	38.6%	
Main countries	Spain	21,087	36.5%	20,009	37.5%	1078	24.5%	
	Italy	5320	9.2%	4509	8.5%	811	18.4%	
	Azerbaijan	4459	7.7%	4235	7.9%	224	5.0%	
	Czeck Republic	477	0.8%	381	0.7%	96	2.2%	

Abbreviations: NSAIDs, non‐steroidal anti‐inflammatory drugs; PPIs, proton pomp inhibitors; Sb, *saccharomyces boulardii* CNCM I‐745 single‐strain; UBT, urea breath test.

^a^ Pearson Chi^2^, two‐sided test if *p* ≥ 0.05 and one‐sided if *p* < 0.05.

^b^ Asymptotic significance (two‐sided).

### 3.2 First‐Line Empirical Prescriptions and Treatment Compliance

Overall prescription patterns varied across European regions: bismuth quadruple‐CAB was most common in the Eastern region (45%), Triple‐CA in Central‐Eastern (59%), single capsule‐MTcB (38%), and concomitant CAM/T (37%) in the South‐West, and sequential‐CAT/M (50%) and single‐capsule MTcB (23%) in Central‐Western. In contrast, the use of Triple‐AM, Triple‐CM, Triple‐AL, and bismuth quadruple MTcB remained consistently low across all regions (< 10% each) (Table S2).

Use of Sb also varied regionally: Eastern—mainly Triple‐CA (38%) and bismuth quadruple CAB (33%); Central‐Eastern—Sb use remained low (< 13%); South‐Western—single capsule‐MTcB (48.4%) and concomitant‐CAM/T (37.4%); Central‐Western—bismuth quadruple single capsule‐MTcB (42%) and Triple‐CA (28%) (Table S2).

**FIGURE 2:**
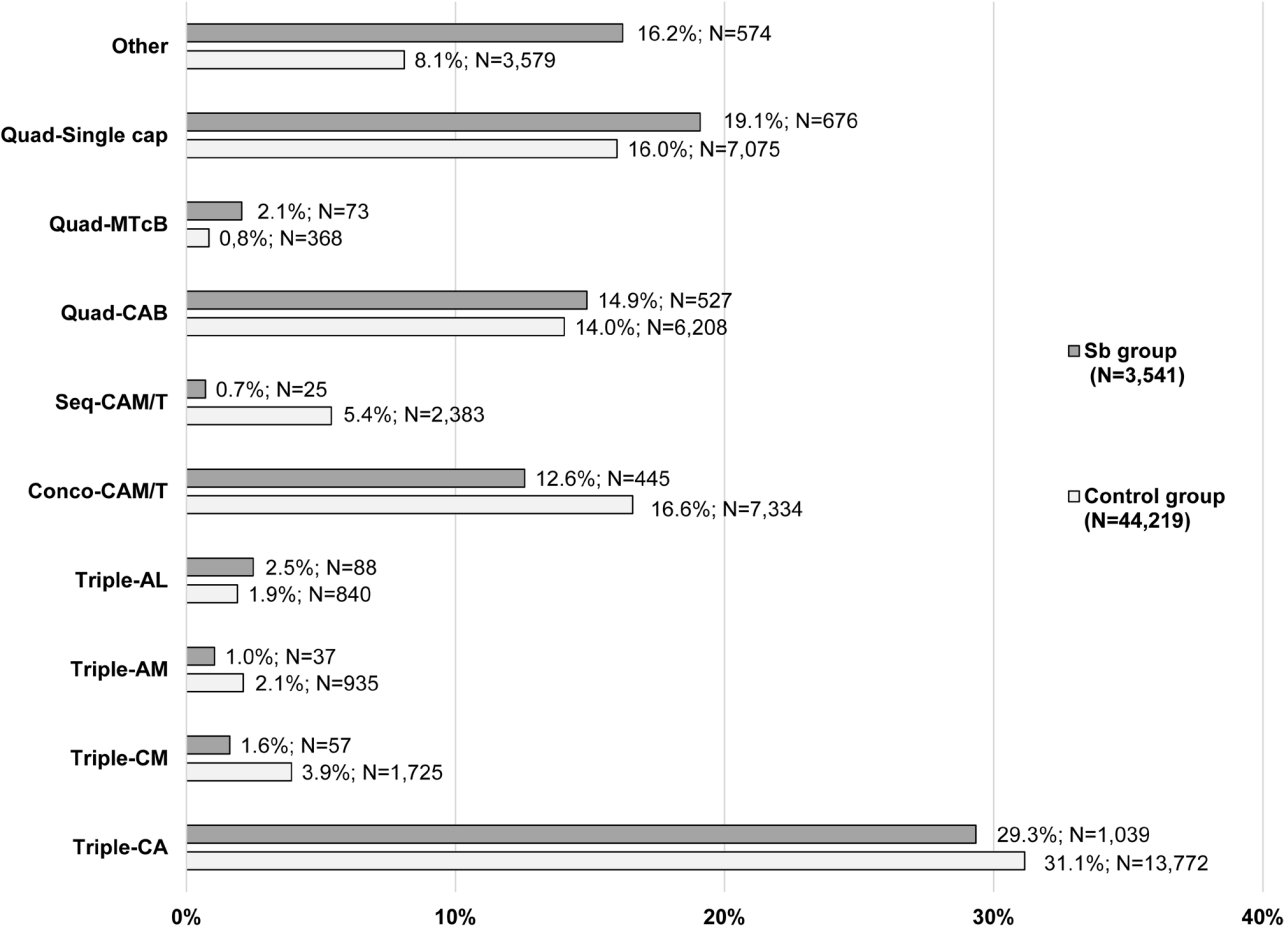
Distribution of the most frequent first‐line empirical therapies in the *S. boulardii* CNCM I‐745 and control groups. A, amoxicillin; B, bismuth salts; C, clarithromycin; Conco, concomitant; L, levofloxacin; M, metronidazole; PPI, proton pomp inhibitor; Quad, quadruple; Sb, *saccharomyces boulardii* CNCM I‐745 (single‐strain); Seq, sequential; T, tinidazole; Tc, tetracycline. Other regimens: PPI + any other antibiotic combination.

At the country level, Sb prescriptions were highest in Russia (39%), Spain (24%), Italy (18%), Azerbaijan (5%), and the Czech Republic (2%). By regimen, Sb was most frequently combined with triple‐CA (29%), single capsule‐MTcB (19%), bismuth quadruple‐CAB (15%), and concomitant‐CAM/T (13%). (Figure 2).

Overall compliance was high (97%; Table S3) and roughly similar between groups (Sb 96.5% vs. control 97%; *p* < 0.05). In Central‐Eastern Europe, compliance was significantly lower in the Sb group, mainly driven by Azerbaijan (control 95% vs. Sb 90%; *p* < 0.01), whereas in Eastern Europe, a significant difference was observed in favor of Sb users, mainly driven by Russia (control 98% vs. Sb 99%; *p* < 0.05). By treatment, no significant differences in compliance were observed except for a slightly higher adherence in Sb users for Triple‐CA (97% vs. 98%, respectively; *p* < 0.05) and the single capsule‐MTcB (97% vs. 95%, respectively; *p* < 0.01).

### 3.3 Sb Impact on Effectiveness

First‐line empirical overall effectiveness was 90%, with no significant difference between the Sb and control groups (both 90%; *p* > 0.05) (Table 2). Multivariate analysis confirmed that adjunctive Sb was not associated with overall effectiveness of first‐line empirical treatment. Across all European regions, mITT overall effectiveness was significantly higher in compliant patients (OR = 10.802; 95% CI: 9.298–12.549), in those receiving standard (OR = 1.453; 1.340–1.576) or high‐dose PPIs (OR = 1.612; 1.474–1.762), and in patients treated for 10 days (OR = 1.544; 1.361–1.752) or 14 days (OR = 1.920; 1.684–2.189) (Table 3). Additionally, female sex, ulcer disease, residence in Eastern or South‐Western Europe, and use of Triple‐AL, concomitant‐CAM/T, or bismuth quadruple single capsule‐MTcB regimens were independently associated with higher eradication success (Table 3a).

**TABLE 2:** Success rates of *H. pylori* eradication therapies with and without *S. boulardii* CNCM I‐745 supplementation.

Success rate		Control group		Sb group		Total		*p*
All countries,	*N* (Total)	38,408		2890		41,298		
all regimens	Success (*N*, %)	34,411	89.6%	2590	89.6%	37,001	89.6%	0.495
**By eradication regimen**								
Triple‐CA	*N* (Total)	10,989		865		11,854		
	Success (*N*, %)	9499	86.4%	751	86.8%	10,250	86.5%	0.396
Triple‐CM	*N* (Total)	1448		52		1500		
	Success (*N*, %)	1229	84.9%	45	86.5%	1274	84.9%	0.465
Triple‐AM	*N* (Total)	865		26		891		
	Success (*N*, %)	748	86.5%	20	76.9%	768	86.2%	0.136
Triple‐AL	*N* (Total)	760		80		840		
	Success (*N*, %)	651	85.7%	74	92.5%	725	86.3%	0.057
Conco‐ CAM/T	*N* (Total)	7064		377		7441		
	Success (*N*, %)	6366	90.1%	352	93.4%	6718	90.3%	0.020
Seq‐ CAM/T	*N* (Total)	2003		23		2026		
	Success (*N*, %)	1790	89.4%	20	87.0%	1810	89.3%	0.451
Quad‐ MTcB	*N* (Total)	353		58		411		
	Success (*N*, %)	307	87.0%	52	89.7%	359	87.3%	0.374
Quad‐Single cap	*N* (Total)	6698		593		7291		
	Success (*N*, %)	6258	93.4%	549	92.6%	6807	93.4%	0.235
Quad‐CAB	*N* (Total)	5284		420		5704		
	Success (*N*, %)	4976	94.2%	385	91.7%	5361	94.0%	0.028
Other regimens	*N* (Total)	2944		396		3340		
	Success (*N*, %)	2587	87.9%	342	86.4%	2929	87.7%	0.217

Abbreviations: A, amoxicillin; B, bismuth salts; C, clarithromycin; Conco, concomitant; L, levofloxacin; M, metronidazole; N, number; PPI, proton pomp inhibitor; Quad, quadruple; Sb, *saccharomyces boulardii;* CNCM I‐745 (single‐strain); Seq, sequential; T, tinidazole; Tc, tetracycline; Triple‐CA: PPI + C + A; Triple‐CM: PPI + C + M; Triple‐AM: PPI + A + M; Triple‐AL: PPI + A + L; Conco‐ CAM/T: Concomitantly PPI + C + A + T or PPI + C + A + M; Seq‐ CAM/T: Alternatively PPI + C + A + T or PPI + C + A + M; Quad‐MTcB: PPI + M + Tc + Bi; Quad‐Single cap: PPI + M + Tc + Bi in a three‐in‐one single capsule formulation; Quad‐CAB: PPI + C + A + B. Other regimens: PPI + any other antibiotic combination.

**TABLE 3:** Multivariate analysis of first‐line empirical overall effectiveness and the impact of *S. boulardii* CNCM I‐745 in European clinical practice.

a. Final model: independent factors associated with overall modified intention‐to‐treat eradication success.					
Europe	Reference	OR	95% CI		*p*‐value^a^
Sex [ref. female]	Female	1.136	1.060	1.217	0.000
Compliance	No (< 90%)	10.802	9.298	12.549	0.000
Indication	Non‐ulcer	1.244	1.134	1.364	0.000
PPI*	Low dose				
Standard dose		1.453	1.340	1.576	0.000
High dose		1.612	1.474	1.762	0.000
Treatment duration	7 days				
10 days		1.544	1.361	1.752	0.000
14 days		1.920	1.684	2.189	0.000
European region	Central‐Western				
Eastern		1.812	1.618	2.029	0.000
Central‐Eastern		0.941	0.848	1.045	0.255
South‐Western		1.829	1.573	2.126	0.000
Eradication regimen	Other regimens**				
Triple‐CA/M		1.111	0.896	1.377	0.337
Triple‐AL		1.739	1.561	1.938	0.000
Conco‐CAM/T		2.820	2.515	3.162	0.000
Quad‐Single cap+Quad‐MTcB		2.159	1.896	2.459	0.000
Quad‐CAB		1.093	0.981	1.218	0.106

b. Final model: independent factors associated with modified intention‐to‐treat eradication success in concomitant CAM/T therapy.^b^					
Concomitant therapy‐CAM/T					
Age		10.009	10.003	1.016	0.005
Compliance	< 90%	9.397	6.124	14.419	0.000
PPI dose**	Low dose				
Standard dose		1.857	1.449	2.379	0.000
High dose		2.427	1.859	3.169	0.000
Treatment duration	7 days				
10 days		3.379	1.374	8.313	0.008
14 days		3.840	1.579	9.337	0.003
Sb use	No	2.322	1.338	4.031	0.003

Abbreviations: A, amoxicillin; B, bismuth salts; C, clarithromycin; Conco, concomitant; CI, confidence interval; L, levofloxacin; M, metronidazole; N, number of patients; OR, odds ratio; PPI, proton pump inhibitor; Quad, quadruple; Sb, *saccharomyces boulardii* CNCM I‐745 (single‐strain); T, tinidazole.

^a^ Statistical significance was set at *p* < 0.05.

* PPI: Proton pump inhibitor; PPI doses: low (4.5–27 mg of omeprazole equivalents given twice a day), standard‐dose (32–40 mg of omeprazole equivalents given twice a day), and high‐dose (54–128 mg of omeprazole equivalents given twice a day).

** Other regimens: any regimen including a PPI+ any other antibiotic combinations than those previously described.

^b^ All models were run for each of the treatment evaluated; however, no significant association was found between the Sb use and a therapy effectiveness increase (data available in Table S5).

### 3.4 Regimen‐Specific Analyses

When analyzed by regimen (Table 2), the Sb significantly improved eradication with concomitant CAM/T (93% vs. 90%; *p* < 0.05; OR = 2.32; 1.34–4.03; *p* < 0.05) (Table 3b) and showed a trend toward higher effectiveness with Triple‐AL (92.5% vs. 86%; *p* ≈0.05). Its use slightly reduced effectiveness with bismuth quadruple CAB (92% vs. 94%; *p* < 0.05), while no significant benefit was observed with bismuth quadruple‐MTcB (90% vs. 87%; *p* > 0.05) and Triple‐CA (87% vs. 86%; *p* > 0.05), or single capsule‐MTcB (93% in both groups; *p* > 0.05); these results were consistent in multivariate analysis (Table 3).

Further information regarding the regimen, regional and country‐specific analyses are reported in File S2, and Tables S4 and S5.

### 3.5 Sb Impact on Safety

Safety data were available for 44,452 patients (3284 in the Sb group and 41,168 controls). Overall, 22% reported at least one AE. The overall incidence of AEs was similar in the Sb group to that in controls (21% vs. 22%; *p* > 0.05; Table S6). A total of 47 serious AEs were documented (7 in Sb and 40 in controls), representing 0.16% and 0.07% in each group, respectively (*p* > 0.05).

Multivariate analysis showed that Sb use was independently associated with a lower risk of experiencing AEs (OR = 0.80; 0.66–0.98; *p* < 0.05), consistently across European regions.

Adherence to therapy (OR = 0.50; 0.41–0.62) and 10‐day prescriptions (OR = 0.77; 0.61–0.98) were significantly associated with fewer AEs (Table 4); whereas, older age (OR = 1.003; 1.001–1.004), ulcer disease (OR = 1.82; 1.65–2.02), standard‐ (OR = 1.43; 1.28–1.60) or high‐dose PPIs use (OR = 1.92; 1.73–2.15), and certain regimens, notably triple‐AL (OR = 3.22; 2.71–3.82) and concomitant CAM/T (OR = 2.43; 2.04–2.89) increased AE risk. Sb users experienced significantly lower rates of AEs including diarrhea (5% vs. 7.5%), dysgeusia (5% vs. 7%), nausea (4% vs. 7%), dyspepsia (2.5% vs. 3%), abdominal pain (2% vs. 4%), asthenia (2% vs. 4%), heartburn (1% vs. 2%), and anorexia (0.3% vs. 3%). No statistically significant difference in vomiting was observed between Sb users and non‐users (2.5% vs. 3%; *p* > 0.05).

**TABLE 4:** Multivariate analysis of safety in first‐line empirical therapy and the impact of *S. boulardii* CNCM I‐745 in European clinical practice.

Dependent variable: incidence of at least one adverse event	Reference category	OR	95% CI		*p*‐value^a^
Age		1.003	1.001	1.004	0.003
Compliance	No (< 90%)	0.505	0.410	0.621	0.000
Indication	Non‐ulcer	1.829	1.655	2.021	0.000
PPI dose*	Low dose				
Standard dose		1.430	1.282	1.594	0.000
High dose		1.930	1.728	2.155	0.000
Treatment duration	7 days				
10 days		0.770	0.607	0.977	0.031
14 days		1.020	0.806	1.290	0.870
Sb use	No	0.802	0.659	0.977	0.028
European region	Eastern				
Central‐Eastern		0.507	0.437	0.589	0.000
South‐Western		0.601	0.509	0.710	0.000
Central‐Western		0.361	0.286	0.454	0.000
Eradication regimen	Other regimens**				
Triple CA/M		1.033	0.720	1.480	0.862
Triple AL		3.219	2.709	3.823	0.000
Conco‐CAM/T		2.424	2.037	2.885	0.000
Quad‐Single cap+Quad‐MTcB		0.687	0.579	0.816	0.000
Quad‐CAB		1.459	1.239	1.718	0.000
**Dependent variable: diarrhea Incidence**					
Age		1.003	1.001	1.004	0.003
Compliance	No (< 90%)	0.505	0.410	0.621	0.000
Indication	Non‐ulcer	1.829	1.655	2.021	0.000
PPI dose*	Low dose				
Standard dose		1.430	1.282	1.594	0.000
High dose		1.930	1.728	2.155	0.000
Treatment duration	7 days				
10 days		0.770	0.607	0.977	0.031
14 days		1.020	0.806	1.290	0.870
Sb use	No	0.803	0.659	0.977	0.028
European region	Eastern				
Central‐Eastern		0.507	0.437	0.589	0.000
South‐Western		0.601	0.509	0.710	0.000
Central‐Western		0.361	0.286	0.454	0.000
Eradication regimen	Triple‐CA/M				
Triple AL		1.033	0.720	1.480	0.862
Conco‐CAM/T		3.219	2.709	3.823	0.000
Quad‐Single cap+Quad‐MTcB		2.424	2.037	2.885	0.000
Quad‐CAB		0.687	0.579	0.816	0.000
Other**		1.459	1.239	1.718	0.000

Abbreviations: A, amoxicillin; B, bismuth salts; C, clarithromycin; Conco, concomitant; CI, confidence interval; L, levofloxacin; M, metronidazole; N, number of patients; OR, odds ratio; PPI, proton pump inhibitor; Quad, quadruple; Sb, *saccharomyces boulardii* CNCM I‐745 (single‐strain); T, tinidazole.

^a^ Statistical significance was set at *p* < 0.05.

* PPI doses: low (4.5–27 mg of omeprazole equivalents given twice a day), standard‐dose (32–40 mg of omeprazole equivalents given twice a day), and high‐dose (54–128 mg of omeprazole equivalents given twice a day).

** Other regimens: any regimen including a PPI+ any other antibiotic combinations than those previously described.

Given that diarrhea was among the most frequently reported AEs (3484 cases) and a condition for which Sb is specifically indicated, we examined this outcome in detail. Multivariate analysis showed that Sb use was associated with a reduced risk of diarrhea (OR = 0.80; 0.66–0.98; *p* < 0.05). Additional factors independently associated with a lower risk of diarrhea included treatment adherence (OR = 0.51; 0.41–0.62), a 7–10‐day treatment duration (OR = 0.77; 0.61–0.98), bismuth quadruple‐CAB (OR = 0.69; 0.58–0.82) and residence in any of the non‐reference European regions. In contrast, increasing age (OR = 1.003; 1.001–1.004), treatment for ulcer disease (OR = 1.83; 1.66–2.02), the use of standard‐ (OR = 1.43; 1.28–1.59) or high‐dose PPIs (OR = 1.93; 1.73–2.16), and several eradication regimens [Conco‐CAM/T (OR = 3.22; 2.71–3.82), single capsule‐MTcB and bismuth quadruple‐MTcB (OR = 2.42; 2.04–2.89), other regimens (OR = 1.46; 1.24–1.72)] were independently associated with a higher risk of diarrhea. The bivariate analysis of safety by region is further specified in File S3 and Table S7.

## 4 Discussion

This large, multi‐country, real‐world study represents the most comprehensive analysis to date of Sb as an adjunct in *H. pylori* eradication therapy across Europe. Sb was the most frequently used single‐strain probiotic in first‐line empirical therapy, and when combined with concomitant‐CAM/T significantly improved eradication success and reduced treatment‐related AEs, particularly gastrointestinal symptoms, such as diarrhea, nausea, dysgeusia, abdominal pain, heartburn, and asthenia. Compliance remained very high (> 95%) across all regimens and countries, with only minimal differences between groups (with vs. without Sb), as reported in previous studies [13, 15, 22].

Regimen‐specific analyses highlighted a significant benefit of Sb in the effectiveness of concomitant CAM/T, a recommended first‐line treatment approach in several geographic regions, suggesting a clinically meaningful adjunctive role [2]. However, benefits were not universal; in bismuth‐containing regimens, no improvement was observed, underscoring the complexity of host–microbe–drug interactions and suggesting that Sb may exert the greatest effect when antibiotic‐induced dysbiosis is pronounced, as is typical with exposure to multiple macrolide–β‐lactam–nitroimidazole combinations. Prior evidence also indicates Sb was associated with significantly lower resistance [15].

Marked geographical variability in outcomes reflected differences in regional prescribing patterns, antibiotic resistance and patient characteristics, with Central‐Eastern and South‐Western regions showing higher eradication success among Sb users than Eastern regions. These findings should be interpreted with caution, as regional prescribing biases and unmeasured confounding—particularly local antibiotic resistance patterns—may contribute to variability. Multivariate analysis confirmed that compliance, high‐potency acid suppression, and longer treatment duration remained the strongest predictors of *H. pylori* eradication success, consistent with international guidelines [1, 23].

Our results confirm that the benefits of probiotic supplementation are regimen‐specific, influenced by regional antibiotic resistance, and support tailored, context‐sensitive clinical recommendations, consistent with prior analyses [24, 25]. Similar reductions in AEs, though smaller effects were observed for eradication outcomes in a recently published Hp‐EuReg study [8], conducting a similar evaluation on the use of different probiotics. Our results also reinforce that eradication success is primarily driven by optimized acid suppression, adequate treatment duration, and high patient compliance, in line with international guidelines [1, 23].

A central finding was the consistent reduction in overall AE incidence among Sb users, particularly diarrhea, supporting its established role in improving tolerability through gut microbiota stabilization, mucosal protection, and inflammation modulation. Maastricht VI (1) specifically mentions Sb as among the strains with the most consistent evidence for reducing therapy‐related AEs. Reduced AEs may further support sustained adherence, particularly in less controlled real‐world settings. Nevertheless, benefits varied by country and regimen, underscoring the influence of antibiotic combinations, PPI dosing, and regional practices.

Strengths of the study include its large sample size, prospective data collection, reflection of European clinical practice, and granular regimen‐ and region‐specific analyses. Limitations include its observational design (and consequent lack of randomization), self‐reported compliance, and relatively small Sb‐treated populations (which subsequently did not allow evaluation of the trends over time).

## 5 Conclusion

In summary, Sb as an adjunct reduced the incidence of AEs and increased effectiveness in specific *H. pylori* eradication regimens, particularly concomitant‐CAM/T therapy. These real‐world findings support the incorporation of Sb into *H. pylori* eradication strategies, reinforcing the value of probiotics in improving tolerability and potentially effectiveness in selected clinical settings.

## Author Contributions

Olga P. Nyssen and Javier P. Gisbert planned and coordinated the study; analyzed, summarized, and interpreted the data, wrote, and reviewed the various drafts of the manuscript and are guarantors of the study. lga P Nyssen, Hp‐EuReg and WorldHpReg Scientific Director, performed the data extraction, supervised the monitoring and the quality check, performed the data analysis, interpretation, and synthesis, critically reviewed the various drafts of the manuscript, and approved the final submission. Javier P. Gisbert, Principal investigator of the registry, directed the project, obtained funding, designed the protocol, recruited patients, assisted with the data analysis, critically reviewed the various drafts of the manuscript, and approved the final submission.

Laimas Jonaitis, Ángeles Pérez‐Aísa, Bojan Tepes, Umud Mahmudov, Irina Voynova, Samuel J. Martínez‐Domínguez, Luis Bujanda, Alfredo J. Lucendo, Ludmila Vologzanina, Ana Garre, Frode Lerang, Sayar R. Abdulkhakov, Matteo Pavoni, Maja Denkovski, Emin Mammadov, Mārcis Leja, Javier Tejedor‐Tejada, Jose M. Huguet, Galyna Fadieienko, Ilchishina Tatiana, Manuel Pabón‐Carrasco, Aiman S. Sarsenbaeva, Oleg Zaytsev, Gülüstan Babayeva, Jesús Barrio, Miguel Areia, Monica Perona, Óscar Núñez, Antonietta G. Gravina, Sergey Alekseenko, Blas José Gómez Rodríguez, Quliyev Fərid Vidadi Oğlu, Inmaculada Ortiz‐Polo, Antonio Moreno Loro, György Miklós Buzás, Boris D. Starostin, Juozas Kupcinskas, Dmitry S. Bordin, Antonio Gasbarrini, Oleksiy Gridnyev, Ricardo Marcos‐Pinto, Manuel Jiménez‐Moreno, Mónica Sánchez Alonso, Virginia Flores, Irene Arteagoitia, Anna Cano‐Català, Pablo Parra, Leticia Moreira, Javier P. Gisbert, collected data, critically reviewed the various drafts of the manuscript, and approved the final submission. Anna Cano and Pablo Parra performed the monitoring and quality check of the data. Leticia Moreira, Pablo Parra, Olga P. Nyssen, Francis Mégraud, Colm O'Morain, Javier P. Gisbert are all members of the Hp‐EuReg and WorldHpReg Scientific Committee; they critically reviewed the various drafts of the manuscript and approved the final submission.

## Funding

This project was promoted and funded by the European Helicobacter and Microbiota Study Group (EHMSG). The Hp‐EuReg was co‐funded by the European Union programme HORIZON (grant agreement number 101095359) and supported by the UK Research and Innovation (grant agreement number 10058099). However, the views and opinions expressed are those of the author(s) only and do not necessarily reflect those of the European Union or the Health and Digitial Executive Agency (HaDEA). Neither the European Union nor the granting authority can be held responsible for them. The Hp‐EuReg was co‐funded by the European Union programme EU4Health (grant agreement number 101101252). The Hp‐EuReg study was partially co‐funded by Mayoly, Diasorin, Juvisé, Biocodex, and Zambon; however, no clinical data were accessible, and the companies were not involved in any stage of the Hp‐EuReg study (design, data collection, and statistical analysis).

## Disclosure

Patient and Public Involvement: Patients and/or the public were not involved in the design, conduct, reporting, or dissemination plans of this research.

## Ethics Statement

The Hp‐EuReg protocol was approved by the Ethics Committee Hospital Universitario de la Princesa (Madrid, SPAIN), which acted as a reference Institutional Review Board (20 December 2012) (Ethics approval code: Hp‐EuReg). This research was conducted according to the guidelines of the Declaration of Helsinki, classified by the Spanish Agency for Medicines and Medical Devices, and prospectively registered at Clinical Trials.gov under the code NCT02328131.

## Conflicts of Interest

Javier P. Gisbert has served as a speaker, consultant, and advisory member for, or has received research funding from: Mayoly, Diasorin, Juvisé, Biocodex, and Zambon. Olga P. Nyssen has served as a speaker or has received research funding from Mayoly, Diasorin, Juvisé, Biocodex, and Zambon. The other authors declare that they have no competing interests.

## Supporting information


**File S1:** Hp‐EuReg investigators.
**File S2:** Impact of S. boulardii CNCM I‐745 use on effectiveness at the regimen, regional and country‐levels.
**File S3:** Safety in the S. boulardii I‐745 group and Control, in each European region.
**Table S1:** List of countries by European geographic region (A) and list of eradication therapies by category (B).
**Table S2:** Use of S. boulardii CNCM I‐745 (Sb) Across First‐Line Eradication Regimens in Europe.
**Table S3:** Compliance by European region, country, and eradication regimen.
**Table S4:** Eradication Success Rates Across European regions, countries and eradication regimen.
**Table S5:** Multivariate analysis of factors associated with eradication effectiveness across H. pylori treatment regimens.
**Table S6:** Overall safety in the Sb and control groups.
**Table S7:** Safety of each treatment by European region.

## Data Availability

Raw data were generated at AEG#x02010; REDCap. All data relevant to the study are included in the article or uploaded as supporting information. De#x02010; identified raw data referring to the current study are available from the WorldHpReg and Hp#x02010; EuReg Scientific director and the principal investigator (PI) of the project (OPN and JPG respectively) upon request. Individual participant data will not be shared.
