# Supplementary material for: Real‐World Effectiveness and Safety of Saccharomyces boulardii CNCM I‐745 as Adjunct Therapy for Helicobacter pylori Eradication: Data From the European Registry on H. pylori Management (Hp‐EuReg)
Source: Helicobacter. 2026 Apr 1;31(2):e70119. doi: 10.1111/hel.70119 (PMC13043801; doi:10.1111/hel.70119)
Supplement: Supplementary file 1 — File S1: Hp‐EuReg investigators. File S2: Impact of S. boulardii CNCM I‐745 use on effectiveness at the regimen, regional and country‐levels. File S3: Safety in the S. boulardii I‐745 group and Control, in each European region. Table S1: List of countries by European geographic region (A) and list of eradication therapies by category (B). Table S2: Use of S. boulardii CNCM I‐745 (Sb) Across First‐Line Eradication Regimens in Europe. Table S3: Compliance by European region, country, and eradication regimen. Table S4: Eradication Success Rates Across European regions, countries and eradication regimen. Table S5: Multivariate analysis of factors associated with eradication effectiveness across H. pylori treatment regimens. Table S6: Overall safety in the Sb and control groups. Table S7: Safety of each treatment by European region. [file HEL-31-e70119-s001.docx]

# SUPPLEMENTARY MATERIAL

**File S1. Hp-EuReg investigators**

Boris D. Starostin, Saint-Petersburg State Budgetary Institution Healthcare City Policlinic 38, Saint-Petersburg, RUSSIA. Acquired data, critically reviewed the manuscript draft, and approved the submitted manuscript.

Sotirios D. Georgopoulos, Athens Medical, P. Faliron General Hospital, Athens, GREECE. Acquired data, critically reviewed the manuscript draft, and approved the submitted manuscript.

Doron Boltin, Division of Gastroenterology, Rabin Medical Center, Faculty of Medicine, Tel Aviv University, Petah Tikva, ISRAEL. Acquired data, critically reviewed the manuscript draft, and approved the submitted manuscript.

Manuel Domínguez Cajal, Department of Gastroenterology and Hepatology, Hospital Universitario San Jorge, Huesca, SPAIN. Acquired data, critically reviewed the manuscript draft, and approved the submitted manuscript.

Galina N. Tarasova, Department of Gastroenterology, Rostov State Medical University, Rostov-on-Don, RUSSIA. Acquired data, critically reviewed the manuscript draft, and approved the submitted manuscript.

Fernando Bermejo, Department of Gastroenterology, Hospital Universitario de Fuenlabrada, Fuenlabrada, SPAIN. Acquired data, critically reviewed the manuscript draft, and approved the submitted manuscript.

Ludmila V. Morkovkina, Republican Gastroenterology Center, Republican Clinical Hospital of the Ministry of Health and Social Development of Chuvashia, Cheboksary, RUSSIA. Acquired data, critically reviewed the manuscript draft, and approved the submitted manuscript.

Cem Simsek, Department of Gastroenterology, Hacettepe University, Mehmet Akif Inan, Health Sciences University, Medical Center, HC International Clinic, Ankara, TURKEY. Acquired data, critically reviewed the manuscript draft, and approved the submitted manuscript.

Perminder S. Phull, Department of Digestive Disorders, Aberdeen Royal Infirmary, Aberdeen, UNITED KINGDOM. Acquired data, critically reviewed the manuscript draft, and approved the submitted manuscript.

María Soledad Marcos, Department of Gastroenterology, Hospital 12 de Octubre, Madrid, SPAIN. Acquired data, critically reviewed the manuscript draft, and approved the submitted manuscript.

Giuseppe Losurdo, Section of Gastroenterology, Department of Precision and Regenerative Medicine and Ionian Area, University of Bari, Bari, ITALY. Acquired data, critically reviewed the manuscript draft, and approved the submitted manuscript.

Judith Gomez-Camarero, Department of Gastroenterology, Hospital Universitario de Burgos, Burgos, SPAIN. Acquired data, critically reviewed the manuscript draft, and approved the submitted manuscript.

Emin Verdiyev, Department of Gastroenterology, Merkezi Klinik Xestexana, Baku, Azerbaijan. Acquired data, critically reviewed the manuscript draft, and approved the submitted manuscript.

Thomas J. Butler, Clinical Medicine, Trinity College Dublin, Department of Gastroenterology, Tallaght University Hospital, Dublin, IRELAND. Acquired data, critically reviewed the manuscript draft, and approved the submitted manuscript.

Sinead M. Smith, School of Medicine, Trinity College Dublin, Dublin, IRELAND. Acquired data, critically reviewed the manuscript draft, and approved the submitted manuscript.

Pedro Almela, Department of Gastroenterology, Hospital General Universitario de Castellón, Ciencias de la Salud (Medicina), Universidad CEU Cardenal Herrera, Castellón, SPAIN. Acquired data, critically reviewed the manuscript draft, and approved the submitted manuscript.

Antonio Mestrovic, Department of Gastroenterology, University Hospital of Split, Split, CROATIA. Acquired data, critically reviewed the manuscript draft, and approved the submitted manuscript.

Natalya N. Dekhnich, Smolensk State Medical University, Smolensk, RUSSIA. Acquired data, critically reviewed the manuscript draft, and approved the submitted manuscript.

Pilar Mata-Romero, Department of Gastroenterology, Hospital Universitario de Cáceres, Cáceres, SPAIN. Acquired data, critically reviewed the manuscript draft, and approved the submitted manuscript.

Daniel Martin-Holgado, Department of Gastroenterology, Hospital Universitario de Cáceres, Cáceres, SPAIN. Acquired data, critically reviewed the manuscript draft, and approved the submitted manuscript.

Marinko Marušić, Department of Gastroenterology, University Hospital Sveti Duh, School of Medicine, University J. J. Strossmayer Osijek, Faculty of Health Studies, University of Rijeka, CROATIA. Acquired data, critically reviewed the manuscript draft, and approved the submitted manuscript.

Ian L. P. Beales, Norwich Medical School, University of East Anglia, Norwich, UNITED KINGDOM. Acquired data, critically reviewed the manuscript draft, and approved the submitted manuscript.

Sabir Sagdati, Department of Gastroenterology and Endoscopy, Gastromedica GM, Novi Pazar, SERBIA. Acquired data, critically reviewed the manuscript draft, and approved the submitted manuscript.

Dmitry N. Andreev, Russian University of Medicine, Moscow, RUSSIA. Acquired data, critically reviewed the manuscript draft, and approved the submitted manuscript.

Igor G. Bakulin, Department of Gastroenterology, Mechnikov North-Western State Medical University, Saint Petersburg, RUSSIA. Acquired data, critically reviewed the manuscript draft, and approved the submitted manuscript.

Ivan Nagorni, Clinical Centre of Nis, Nis, SERBIA. Acquired data, critically reviewed the manuscript draft, and approved the submitted manuscript.

Alla Kononova, Tver State Madical University, Tver, RUSSIA. Acquired data, critically reviewed the manuscript draft, and approved the submitted manuscript.

Vladimir Milivojevic, Department of Gastroenterology and Hepatology, University Clinical Centre of Serbia, Belgrade University, Belgrade, SERBIA. Acquired data, critically reviewed the manuscript draft, and approved the submitted manuscript.

Noelia Alcaide, Department of Gastroenterology, Hospital Clínico de Valladolid, Valladolid, SPAIN. Acquired data, critically reviewed the manuscript draft, and approved the submitted manuscript.

Benito Velayos, Department of Gastroenterology, Hospital Clínico de Valladolid, Valladolid, SPAIN. Acquired data, critically reviewed the manuscript draft, and approved the submitted manuscript.

Luis Fernández-Salazar, Department of Gastroenterology, Hospital Clínico de Valladolid, Medicine Department, School of Medicine, Universidad de Valladolid, Valladolid, SPAIN. Acquired data, critically reviewed the manuscript draft, and approved the submitted manuscript.

Georges Kamtoh, Hepatic Medical, Private Medical Center, Krakow, POLAND. Acquired data, critically reviewed the manuscript draft, and approved the submitted manuscript.

Eduardo Iyo, Department of Gastroenterology, Hospital Universitari Son Espases, Palma (Mallorca), SPAIN. Acquired data, critically reviewed the manuscript draft, and approved the submitted manuscript.

Pablo M. Wolfe García, Department of Gastroenterology and Hepatology, Hospital Sierrallana, Torrelavega, SPAIN. Acquired data, critically reviewed the manuscript draft, and approved the submitted manuscript.

Natalia V. Bakulina, Department of Gastroenterology, Mechnikov North-Western State Medical University, Saint Petersburg, RUSSIA. Acquired data, critically reviewed the manuscript draft, and approved the submitted manuscript.

Ramón Pajares Villarroya, Gastroenterology Section, Hospital Universitario Infanta Sofía, Facultad de Medicina, Universidad Europea de Madrid, San Sebastián de los Reyes, SPAIN. Acquired data, critically reviewed the manuscript draft, and approved the submitted manuscript.

Miguel Fernández-Bermejo, Department of Gastroenterology, Hospital Parque San Francisco, Cáceres, SPAIN. Acquired data, critically reviewed the manuscript draft, and approved the submitted manuscript.

Jurij Bednarik, Department of Gastroenterology, Klinika Doktor 24 Ljubljana, SLOVENIA. Acquired data, critically reviewed the manuscript draft, and approved the submitted manuscript.

Debora Compare, Department of Gastroenterology, Federico II University, Naples, ITALY. Acquired data, critically reviewed the manuscript draft, and approved the submitted manuscript.

Sabina Hrubá, 1st internal clinic, Department of gastroenterology and hepatology, University hospital in Pilsen, Pilsen, CZECH REPUBLIC. Acquired data, critically reviewed the manuscript draft, and approved the submitted manuscript.

Marina F. Osipenko, Novosibirsk State Medical University, Ministry of Health of Russia, Novosibirsk, RUSSIA. Acquired data, critically reviewed the manuscript draft, and approved the submitted manuscript.

Dan L. Dumitrascu, Iuliu Hatieganu University of Medicine and Pharmacy, Cluj-Napoca, ROMANIA. Acquired data, critically reviewed the manuscript draft, and approved the submitted manuscript.

Alisan Kahraman, Gastroenterology and Hepatology Department, Max Grundig Clinic, Bühl/Baden, GERMANY. Acquired data, critically reviewed the manuscript draft, and approved the submitted manuscript.

Emilija Nikolovska Trpchevska, University Clinic for Gastroenterohepatology, Faculty of Medicine, Ss. Cyril and Methodius University in Skopje, Skopje, Republic of North Macedonia. Acquired data, critically reviewed the manuscript draft, and approved the submitted manuscript.

Montserrat Planella, Department of Gastroenterology, Hospital Universitari Arnau de Vilanova, Institut de Recerca Biomèdica de Lleida (IRBLL), Lleida, SPAIN. Acquired data, critically reviewed the manuscript draft, and approved the submitted manuscript.

Consuelo Ramírez, Department of Gastroenterology, Hospital Universitari Arnau de Vilanova, Institut de Recerca Biomèdica de Lleida (IRBLL), Lleida, SPAIN. Acquired data, critically reviewed the manuscript draft, and approved the submitted manuscript.

Victor A. Kamburov, Department of Gastroenterology, BalkanMed Medical Center, Sofia, BULGARIA. Acquired data, critically reviewed the manuscript draft, and approved the submitted manuscript.

Teresa Angueira, Department of Gastroenterology, Hospital Universitario de Toledo, Toledo, SPAIN. Acquired data, critically reviewed the manuscript draft, and approved the submitted manuscript.

Natalia V. Baryshnikova, Pavlov First Saint Petersburg State Medical University, Saint Petersburg, RUSSIA. Acquired data, critically reviewed the manuscript draft, and approved the submitted manuscript.

Ana Beatriz Pozo Blanco, Department of Gastroenterology, Hospital Arnau Vilanova-Lliria, Valencia, SPAIN. Acquired data, critically reviewed the manuscript draft, and approved the submitted manuscript.

Pedro Delgado Guillena, Department of Gastroenterology, Hospital de Mérida, Mérida, SPAIN. Acquired data, critically reviewed the manuscript draft, and approved the submitted manuscript.

Maria A. Livzan, Omsk State Medical University, Omsk, RUSSIA. Acquired data, critically reviewed the manuscript draft, and approved the submitted manuscript.

Melanija Razov Radas, Department of Gastroenterology and Hepatology, General Hospital Zadar, Health Studies, University of Zadar, Zadar, CROATIA. Acquired data, critically reviewed the manuscript draft, and approved the submitted manuscript.

Natalya V. Bakanova, Medical Center “Medicea”, Izhevsk, RUSSIA. Acquired data, critically reviewed the manuscript draft, and approved the submitted manuscript.

Eva Barreiro Alonso, Department of Gastroenterology, Hospital Central de Asturias (HUCA), Department of Pharmacology, Instituto de Investigación Sanitaria del Principado de Asturias (ISPA), Oviedo, SPAIN. Acquired data, critically reviewed the manuscript draft, and approved the submitted manuscript.

Michael Doulberis, Department of Gastroenterology, Kantonsspital Aarau, Aarau, Switzerland. Acquired data, critically reviewed the manuscript draft, and approved the submitted manuscript.

Rosario Antón Ausejo, Department of Gastroenterology, Hospital Clínico Universitario de Valencia, Valencia, SPAIN. Acquired data, critically reviewed the manuscript draft, and approved the submitted manuscript.

Piotr Eder, Department of Gastroenterology, Dietetics and Internal Medicine, Poznań University of Medical Sciences, Department of Gastroenterology, H. Święcicki University Hospital, Poznan, POLAND. Acquired data, critically reviewed the manuscript draft, and approved the submitted manuscript.

Natasa Brglez Jurecic, Interni Oddelek, Diagnostic Centre, Bled, SLOVENIA. Acquired data, critically reviewed the manuscript draft, and approved the submitted manuscript.

Wojciech Marlicz, Department of Gastroenterology, Pomeranian Medical University in Szczecin, The Centre for Digestive Diseases, Endoklinika, Szczecin, POLAND. Acquired data, critically reviewed the manuscript draft, and approved the submitted manuscript.

Sheyla Montori Pina, Department of Gastroenterology, Hospital Universitario de Navarra (HUN), Navarrabiomed, Universidad Pública de Navarra (UPNA), IdiSNA, Pamplona, SPAIN. Acquired data, critically reviewed the manuscript draft, and approved the submitted manuscript.

Antonio Cuadrado, Department of Gastroenterology and Hepatology, Hospital Universitario Marqués de Valdecilla, Clinical and Translational Research in Digestive Diseases, Valdecilla Research Institute (IDIVAL), Santander, SPAIN. Acquired data, critically reviewed the manuscript draft, and approved the submitted manuscript.

Jan Kral, Medic Kral s.r.o., Praha, CZECH REPUBLIC. Acquired data, critically reviewed the manuscript draft, and approved the submitted manuscript.

Francisco J. Rancel-Medina, Department of Gastroenterology, Complejo Asistencial Universitario de Palencia, Palencia, SPAIN. Acquired data, critically reviewed the manuscript draft, and approved the submitted manuscript.

Christos Liatsos, Gastroenterology Department, 401 Military Hospital of Athens, Athens, GREECE. Acquired data, critically reviewed the manuscript draft, and approved the submitted manuscript.

Olga A. Kolokolnikova, Clinical Hospital №1 MEDSI, Moscow, RUSSIA. Acquired data, critically reviewed the manuscript draft, and approved the submitted manuscript.

Manon C.W. Spaander, Department of Gastroenterology and Hepatology, Erasmus University Medical Center, Rotterdam, THE NETHERLANDS. Acquired data, critically reviewed the manuscript draft, and approved the submitted manuscript.

Piotr Szredzki, The John Paul II City Hospital, Rzeszow, POLAND. Acquired data, critically reviewed the manuscript draft, and approved the submitted manuscript.

María Badía Martínez, Department of Gastroenterology, Hopital General de la Defensa, Department of Gastroenterology, Hospital Universitario Miguel Servet, Zaragoza, SPAIN. Acquired data, critically reviewed the manuscript draft, and approved the submitted manuscript.

Stergios N. Kouvaras, Refferal Endoscopy Unit, Private Endoscopy Unit, Halkida, GREECE. Acquired data, critically reviewed the manuscript draft, and approved the submitted manuscript.

Mila Kovacheva-Slavova, Department of Gastroenterology, Queen Yoanna University Hospital, Medical University of Sofia, Sofia, BULGARIA. Acquired data, critically reviewed the manuscript draft, and approved the submitted manuscript.

Regina I. Khlynova, Department of faculty therapy and geriatrics, Ural State Medical University, Ekaterinburg, RUSSIA. Acquired data, critically reviewed the manuscript draft, and approved the submitted manuscript.

Benito Hermida Pérez, Department of Gastroenterology, Hospital Valle del Nalón, Langreo, SPAIN. Acquired data, critically reviewed the manuscript draft, and approved the submitted manuscript.

Petra Čavajdová, University Hospital Hradec Kralove, Hradec Kralove, CZECH REPUBLIC. Acquired data, critically reviewed the manuscript draft, and approved the submitted manuscript.

Sergio Gil Rojas, Department of Gastroenterology, Hospital Virgen de la Luz, Cuenca, SPAIN. Acquired data, critically reviewed the manuscript draft, and approved the submitted manuscript.

Luis Hernández, Department of Gastroenterology, Hospital Santos Reyes, Aranda de Duero, SPAIN. Acquired data, critically reviewed the manuscript draft, and approved the submitted manuscript.

Ekaterina Y. Plotnikova, Department of Polyclinic Therapy, Postgraduate Training of Doctors and Nursing, Federal State Higher Education Institution Kemerovo State Medical University Ministry of Health of Russia, Kemerovo, RUSSIA. Acquired data, critically reviewed the manuscript draft, and approved the submitted manuscript.

Xhensila Pemaj, Department of Gastroenterology and Internal Medicine, Amerikan Hospital Albania, Tirana, ALBANIA. Acquired data, critically reviewed the manuscript draft, and approved the submitted manuscript.

Deirdre McNamara, Trinity Academic Gastroenterology Group (TAGG) Research Centre, School of Medicine, Trinity College Dublin, Department of Gastroenterology, Tallaght University Hospital, Dublin, IRELAND. Acquired data, critically reviewed the manuscript draft, and approved the submitted manuscript.

Guillem Soy, Department of Gastroenterology, Hospital Clínic de Barcelona, Institut Clínic de Malalties Digestives i Metabòliques (ICMDM), Barcelona, SPAIN. Acquired data, critically reviewed the manuscript draft, and approved the submitted manuscript.

Ioannis Linas, Department of Gastroenterology, GGP Bern, Department of Gastroenterology, Hirslanden Klinik Beau-Site, Bern, Switzerland. Acquired data, critically reviewed the manuscript draft, and approved the submitted manuscript.

Riccardo Vasapolli, Medical Department 2, University Hospital LMU Munich, Munich, GERMANY Acquired data, critically reviewed the manuscript draft, and approved the submitted manuscript.

Marko Nikolic, University Centre Sestre Milosrdnice, Zagreb, CROATIA. Acquired data, critically reviewed the manuscript draft, and approved the submitted manuscript.

Andreas Blesl, Department of Internal Medicine, Division of Gastroenterology and Hepatology, Medical University of Graz, Graz, AUSTRIA. Acquired data, critically reviewed the manuscript draft, and approved the submitted manuscript.

Tamara Matysiak-Budnik, Hepato-Gastroenterology & Digestive Oncology Unit, University Hospital of Nantes, Nantes, FRANCE. Acquired data, critically reviewed the manuscript draft, and approved the submitted manuscript.

Diego Burgos-Santamaría, Department of Gastroenterology and Hepatology, Hospital Universitario Ramón y Cajal, Madrid, SPAIN. Acquired data, critically reviewed the manuscript draft, and approved the submitted manuscript.

Rashad A. Hasanov, Department of Gastroenterology, German Hospital, Baku, Azerbaijan. Acquired data, critically reviewed the manuscript draft, and approved the submitted manuscript.

Lumir Kunovsky, 2nd Department of Internal Medicine - Gastroenterology and Geriatrics, University Hospital Olomouc, Faculty of Medicine and Dentistry, Palacky University Olomouc, Department of Surgery, University Hospital Brno, Faculty of Medicine, Masaryk University, Department of Gastroenterology and Digestive Endoscopy, Masaryk Memorial Cancer Institute, CZECH REPUBLIC. Acquired data, critically reviewed the manuscript draft, and approved the submitted manuscript.

Carlos Maroto-Martín, Department of Gastroenterology, Hospital Río Hortega de Valladolid, Valladolid, SPAIN. Acquired data, critically reviewed the manuscript draft, and approved the submitted manuscript.

Pilar Bernal Checa, Department of Gastroenterology, Hospital Universitario Miguel Servet, Instituto de Investigación Sanitaria de Aragón (IIS Aragón), Zaragoza, SPAIN. Acquired data, critically reviewed the manuscript draft, and approved the submitted manuscript.

Paola Chaudarcas, Department of Gastroenterology, Hospital Universitario Infanta Sofía, San Sebastián de los Reyes, SPAIN. Acquired data, critically reviewed the manuscript draft, and approved the submitted manuscript.

Pilar Pazo Mejide, Department of Gastroenterology, Hospital de Cruces, Barakaldo, SPAIN. Acquired data, critically reviewed the manuscript draft, and approved the submitted manuscript.

Giulia Fiorini, Cardiovascular Medicine Unit, Heart, Chest and Vascular Department, IRCCS Azienda Ospedaliero-Universitaria di Bologna, Hypertension and Cardiovascular Risk Research Center, Medical and Surgical Sciences Dept., Alma Mater Studiorum University of Bologna, Bologna, ITALY. Acquired data, critically reviewed the manuscript draft, and approved the submitted manuscript.

Ramiro Carreño Macián, Department of Gastroenterology, Hospital Quirónsalud Vitoria, Vitoria, SPAIN. Acquired data, critically reviewed the manuscript draft, and approved the submitted manuscript.

Rosa Rosania, Department of Gastroenterology, Hepatology and Infectious Diseases, Otto von Guericke University Hospital, Magdeburg, GERMANY. Acquired data, critically reviewed the manuscript draft, and approved the submitted manuscript.

Anna-Maria Tiefenthaller, Department of Gastroenterology, Barmherzige Schwestern Linz, Linz, AUSTRIA. Acquired data, critically reviewed the manuscript draft, and approved the submitted manuscript.

Teresa Valdés-Lacasa, Department of Gastroenterology, Hospital Universitario Infanta Cristina, Parla, SPAIN. Acquired data, critically reviewed the manuscript draft, and approved the submitted manuscript.

Amir Mari, Gastroenterology and Endoscopy Unit, Nazareth Hospital EMMS, Faculty of Medicine, Nazareth, Israel, Bar-Ilan University, ISRAEL. Acquired data, critically reviewed the manuscript draft, and approved the submitted manuscript.

Anna L. Pakhomova, V. I. Razumovsky Saratov state medical University, Saratov, RUSSIA. Acquired data, critically reviewed the manuscript draft, and approved the submitted manuscript.

Jan Bornschein Nuffield Department of Experimental Medicine, University of Oxford, Oxford, UNITED KINGDOM. Acquired data, critically reviewed the manuscript draft, and approved the submitted manuscript.

Suzanne Cauchi, Mater Dei Hospital, Msida, MALTA. Acquired data, critically reviewed the manuscript draft, and approved the submitted manuscript.

Jesus M. Gonzalez-Santiago, Department of Gastroenterology, Complejo Asistencial Universitario de Salamanca, Instituto de Investigación Biomédica de Salamanca (IBSAL), Centro de Investigación Biomédica en Red de Enfermedades Hepáticas y Digestivas (CIBERehd), Salamanca, SPAIN. Acquired data, critically reviewed the manuscript draft, and approved the submitted manuscript.

Petra Koňaříková, Tomas Bata Regional Hospital, Zlin, CZECH REPUBLIC. Acquired data, critically reviewed the manuscript draft, and approved the submitted manuscript.

Isabel Pérez-Martínez, Department of Gastroenterology, Hospital Universitario Central de Asturias, Instituto de Investigación Sanitaria del Principado de Asturias (ISPA), Oviedo, SPAIN. Acquired data, critically reviewed the manuscript draft, and approved the submitted manuscript.

David Přidal, Department of Gastroenterology, SPEA Olomouc, Olomouc, CZECH REPUBLIC. Acquired data, critically reviewed the manuscript draft, and approved the submitted manuscript.

Jorge Yebra Carmona, Department of Gastroenterology, Hospital Universitario de Móstoles, Móstoles, SPAIN. Acquired data, critically reviewed the manuscript draft, and approved the submitted manuscript.

Miguel Suárez Matías, Department of Gastroenterology, Hospital Virgen de la Luz, Cuenca, SPAIN. Acquired data, critically reviewed the manuscript draft, and approved the submitted manuscript.

Natalie Friedova, Department of Gastroenterology, Thomayer University Hospital, Prague, CZECH REPUBLIC. Acquired data, critically reviewed the manuscript draft, and approved the submitted manuscript.

Diego Ledro Cano, Department of Gastroenterology, Clínica HLA Santa Isabel, Department of Gastroenterology, Hospital Universitario Virgen Macarena, Sevilla, SPAIN. Acquired data, critically reviewed the manuscript draft, and approved the submitted manuscript.

Mirjana Kalauz, Endoscopy Unit, Division of Gastroenterology, Department of Internal Medicine, Clinical Hospital Center Zagreb, Internal Medicine, School of Medicine, University of Zagreb, Zagreb, CROATIA. Acquired data, critically reviewed the manuscript draft, and approved the submitted manuscript.

Jose Xavier Segarra Ortega, Department of Gastroenterology, Hospital Universitario de Salamanca, Grupo de Investigación Salmantino en Aparato Digestivo (GISAD), Instituto de Investigación Biomédica de Salamanca, Salamanca, SPAIN. Acquired data, critically reviewed the manuscript draft, and approved the submitted manuscript.

Adam Vasura, Department of Gastroenterology, Hepatology and Pancreatology, Internal and Cardiologic Clinic, University Hospital of Ostrava, Ostrava, CZECH REPUBLIC. Acquired data, critically reviewed the manuscript draft, and approved the submitted manuscript.

Senador Moran Sanchez, Department of Gastroenterology, Servicio Murciano de Salud, Cartagena, SPAIN. Acquired data, critically reviewed the manuscript draft, and approved the submitted manuscript.

Petr Bauer, Department of Gastroenterology, Hospital Děčín, Děčín, CZECH REPUBLIC. Acquired data, critically reviewed the manuscript draft, and approved the submitted manuscript.

Katarina Jankovic, Clinic for Gastroenterohepatology, University Clinical Centre of Serbia, Belgrade, SERBIA. Acquired data, critically reviewed the manuscript draft, and approved the submitted manuscript.

Sara Hoxha, University Hospital Center "Mother Teresa", Tirana, ALBANIA. Acquired data, critically reviewed the manuscript draft, and approved the submitted manuscript.

Leticia Gimeno Pitarch, Department of Gastroenterology, Hospital General Universitario de Castellón, Castellón, SPAIN. Acquired data, critically reviewed the manuscript draft, and approved the submitted manuscript.

Marjan Stankovic, Clinical Hospital Center Zemun, Belgrade, SERBIA. Acquired data, critically reviewed the manuscript draft, and approved the submitted manuscript.

Hagai Schweistein, Department of Gastroenterology and Hepatology, Rabin Medical Center, Petah Tikva, ISRAEL. Acquired data, critically reviewed the manuscript draft, and approved the submitted manuscript.

Alma Keco-Huerga, Department of Gastroenterology, Hospital Universitario Virgen Macarena, Sevilla, SPAIN. Acquired data, critically reviewed the manuscript draft, and approved the submitted manuscript.

Ismar Hasukić, Department of Gastroenterology and Hepatology, University Clinical Center Tuzla, Tuzla, Bosnia and Herzegovina. Acquired data, critically reviewed the manuscript draft, and approved the submitted manuscript.

Thomas Balanis, Department of Gastroenterology, Kantonspital Schaffhausen, Zürich, Switzerland. Acquired data, critically reviewed the manuscript draft, and approved the submitted manuscript.

Jakub Langner, Beskydy Gastrocentre, Hospital Frydek-Mistek, Frydek-Mistek, CZECH REPUBLIC. Acquired data, critically reviewed the manuscript draft, and approved the submitted manuscript.

Patrick Dinkhauser, I. Interne Abteilung, Klinikum Wels-Grieskirchen, Wels, AUSTRIA. Acquired data, critically reviewed the manuscript draft, and approved the submitted manuscript.

Patricia Sanz-Segura, Department of Gastroenterology, Hospital Royo Villanova, Zaragoza, SPAIN. Acquired data, critically reviewed the manuscript draft, and approved the submitted manuscript.

Melek Balamir, Department of Gastroenterology, Cerrahpasa Medical Faculty, Istanbul University Cerrahpasa, Istanbul, Turkey Acquired data, critically reviewed the manuscript draft, and approved the submitted manuscript.

Theodore Rokkas, Department of Gastroenterology, Henry Dunant Hospital, Athens, GREECE. Acquired data, critically reviewed the manuscript draft, and approved the submitted manuscript.

Mikel Ganuza, Department of Gastroenterology, Hospital General de Granollers, Barcelona, SPAIN. Acquired data, critically reviewed the manuscript draft, and approved the submitted manuscript.

Milica Bjelakovic, Klinika za gastroenterologiju i hepatologiju, Univerzitetski klinicki centar Nis, Nis, SERBIA Acquired data, critically reviewed the manuscript draft, and approved the submitted manuscript.

Antonia Perelló, Department of Gastroenterology, Hospital Universitari Son Espases, Palma (Mallorca), SPAIN. Acquired data, critically reviewed the manuscript draft, and approved the submitted manuscript.

Marta Pascual-Mato, Department of Gastroenterology and Hepatology, Clinical and Translational Research in Digestive Diseases, Valdecilla Research Institute (IDIVAL), Marqués de Valdecilla University Hospital, Santander, SPAIN. Acquired data, critically reviewed the manuscript draft, and approved the submitted manuscript.

Alexander Link, Friedrich-Alexander-Universität Erlangen-Nürnberg (FAU), Medizincampus Oberfranken, Klinikum Bayreuth GmbH, Bayreuth, GERMANY. Acquired data, critically reviewed the manuscript draft, and approved the submitted manuscript.

Marino Venerito, Department of Gastroenterology, Hepatology and Infectious Diseases, University Hospital of Magdeburg, Magdeburg, GERMANY. Acquired data, critically reviewed the manuscript draft, and approved the submitted manuscript.

Jan Krivinka, 2nd Department of Internal Medicine – Gastroenterology and Geriatrics, University Hospital Olomouc, Faculty of Medicine and Dentistry, Palacky University Olomouc, Olomouc, Czech Republic. Acquired data, critically reviewed the manuscript draft, and approved the submitted manuscript.

Cristina Maria Sabo, 2nd Medical Dept, Iuliu Hațieganu University of Medicine and Pharmacy, Cluj Napoca, ROMANIA. Acquired data, critically reviewed the manuscript draft, and approved the submitted manuscript.

Lyudmila Boyanova, Department of Medical Microbiology, Medical University of Sofia, Sofia, BULGARIA. Acquired data, critically reviewed the manuscript draft, and approved the submitted manuscript.

Eduardo Albéniz, Department of Gastroenterology, Hospital Universitario de Navarra (HUN), Navarrabiomed, Universidad Pública de Navarra (UPNA), IdiSNA, Pamplona, SPAIN. Acquired data, critically reviewed the manuscript draft, and approved the submitted manuscript.

Skerdi Prifti, Department of Gastroenterology, University Hospital Center Mother Teresa, Tirana, ALBANIA Acquired data, critically reviewed the manuscript draft, and approved the submitted manuscript.

Pierre Ellul, Department of Gastroenterology, Mater Dei Hospital, Msida, MALTA. Acquired data, critically reviewed the manuscript draft, and approved the submitted manuscript.

Katja Repitsch, Department of Gastroenterology, Klinikum Klagenfurt am Wörthersee, Klagenfurt, AUSTRIA. Acquired data, critically reviewed the manuscript draft, and approved the submitted manuscript.

Ramazan Erdem Er, Ankara University Faculty of Medicine, Ankara, TURKEY. Acquired data, critically reviewed the manuscript draft, and approved the submitted manuscript.

Skender Telaku, University for Business and Technology, Pristina, KOSOVO Acquired data, critically reviewed the manuscript draft, and approved the submitted manuscript.

**File S2. Impact of S. boulardii CNCM I-745 use on effectiveness at the regimen, regional and country-levels**

In these regimens (Table S4), compliance emerged as the strongest independent predictor of eradication success, with non-adherent patients showing markedly reduced effectiveness, particularly in standard triple therapy (OR ~19) and single-capsule bismuth therapy (OR ~14). PPI potency was also consistently associated with improved outcomes, with both standard- and high-dose PPIs outperforming low-dose regimens in all models. In the bismuth-based models, rebamipide use substantially increased eradication success (OR ~5.7). For standard triple therapy, longer treatment durations (10–14 days) significantly improved success compared with 7-day regimens. Regional effects were heterogeneous: Eastern and South-Western Europe demonstrated higher odds of success in standard triple therapy, while no region showed a statistically significant effect in the Single capsule regimen.

*Regional Analyses:*

Analysis by geographical region revealed heterogeneity in treatment outcomes among the four areas where *Sb* is marketed. When examining eradication rates directly, statistically significant differences favoring Sb users were observed in the Central-Eastern region (97% vs 90%; *p*<0.001), largely driven by results from Azerbaijan (97% vs 93%; *p*<0.05) and the Czech Republic (94% vs 85.5%; *p*<0.05). A smaller but significant benefit was also seen in the South-Western region (92% vs 89%; *p*<0.01), mainly attributable to outcomes in Spain (92% vs 89%; *p*<0.01). Conversely, no significant difference was observed between groups in the Central-Western region (*p*>0.05). In the Eastern region, eradication rates were significantly lower among Sb users (87% vs 90%; *p*<0.01), a finding primarily driven by results from Russia (87% vs 90%; *p*<0.001). In the logistic regression model, both the Central-Eastern (OR = 1.81; 95% CI: 1.62–2.03, *p*<0.001) and Central- Western (OR = 1.83; 95% CI: 1.57–2.13 *p*<0.001) regions showed significantly higher odds of the outcome compared with the Eastern reference region, whereas the South-Western region (OR = 0.94; 95% CI: 0.85–1.05; p=0.25) did not differ significantly.

*Country-Specific Analyses:*

Comparable heterogenous results emerged when treatment effectiveness was assessed within the principal countries contributing to regional prescribing trends (Table S4).

For instance, in Spain, the addition of *Sb* to the concomitant CAM/T regimen significantly increased eradication rates (95% vs 89%; p<0.001), and this benefit remained significant in the logistic regression analysis (OR = 2.12; 95% CI: 1.18–3.80; p<0.05). Within the quadruple-CAM regimen, treatment success was consistently driven by compliance (OR = 9.191; 95% CI: 5.782–14.609; p<0.0001) and PPI potency (standard: OR = 1.638; 95% CI: 1.261–2.129; p<0.0001; high: OR = 2.647; 95% CI: 1.894–3.700; p<0.0001). No significant benefit of Sb was observed when combined with Triple-CA (81% vs 81%; 25 vs 1,681; p>0.05), single capsule-MTcB (91.5% vs 93%; p>0.05), or bismuth quadruple CAB (83% vs 91%; p>0.05). For the single capsule-MTcB regimen, eradication outcomes were primarily determined by compliance (OR = 13.354; 95% CI: 8.776–20.321; p<0.0001) and PPI potency (intermediate: OR = 1.849; 95% CI: 1.438–2.379; p<0.0001; high: OR = 2.195; 95% CI: 1.677–2.872; p<0.0001).

In Italy**,** there was a trend toward higher eradication rates when Sb was combined with Triple-CA (82% vs 78%; p>0.05), and lower rates when used with the single capsule-MTcB (94% vs 96%; p>0.05) or concomitant-CAM/T therapy (83% vs 96%; p≈0.05). However, in the multivariate analysis, after adjustment for relevant covariates, neither Sb use nor other tested variables (age, sex, PPI dose) remained in the final model. Two independent predictors of treatment success were identified: Compliance was the strongest determinant, with compliant patients showing markedly higher odds of eradication (OR = 24.08; 95% CI: 8.69–66.68; p<0.001). Conversely, ulcer-related indication was associated with reduced effectiveness (OR = 0.33; 95% CI: 0.12–0.91; p<0.05).

In the Czech Republic, Sb added to triple-CA was significantly associated with higher eradication rate (98% vs 84%; 41 vs 147; p<0.05), only in those patients that had been compliant with therapy. This effect, however, was not confirmed in the multivariate logistic regression analysis (p>0.05).

In Russian patients, and across both triple-CA and bismuth quadruple-CAB regimens, the use of Sb was not associated with improved eradication rates. Other clinical factors demonstrated significant and consistent contributions to treatment success in the multivariable logistic regression model. These included compliance (OR = 9.39; 5.07–17.40; p<0.001), PPI potency (intermediate: OR = 1.48; 1.08–2.04; p<0.05; high: OR = 2.70; 95% CI:1.62–4.50; p<0.001), and treatment duration (14 days: OR = 3.42; 1.48–7.90; p<0.01).

In Azerbaijan, significant higher eradication rates were observed when Sb was combined with triple-CA (97% vs 91.5%; p<0.05); however, this effect was not confirmed in the multivariate logistic regression analysis (p>0.05). Higher eradication rates were also observed when Sb was combined with triple-CM (100% vs 94%), triple-AL (95% vs 91.5%), and bismuth quadruple-CAB (100% vs 99%), but the differences were not significant (p>0.05).

**File S3. Safety in the S. boulardii I-745 group and Control, in each European region**

When stratified by the most frequently prescribed treatments in each region (Table S6), compared to the control group, the incidence of AEs significantly decreased in the Eastern region uniquely when Sb was used with Quad-CAB (70% vs 77%; p<0.05) but increased when prescribed with Triple-CA (30% vs 23%; p<0.05); however, it decreased in Central-Eastern region with Triple-CA (25% vs 61%; p<0.001) but not with triple-AL (75% vs 39%; p<0.001). Additionally, in the South-Western region, Sb use significantly decreases the incidence of AEs when added to the Quad-CAM/T (15% vs 42%; p<0.001), and it conversely increased with the single capsule-MTcB (85% vs 58%; p<0.001). However, in the Central-Western region the incidence was reduced when Sb was combined with the single capsule-MTcB (86% vs 99%; p<0.001) and again increased when combined with Triple-CA (14% vs 0.7%; p<0.001).

**Table S1: List of countries by European geographic region (A) and list of eradication therapies by category (B)**

| **(A) European regions and countries** | | |
| --- | --- | --- |
|  | Eastern | Albania, Bulgaria, Romania, Russia, Ukraine, Turkey, |
|  | Central-Eastern | Azerbaijan, Czech Rep, Greece, Hungary, Latvia, Lithuania, Malta, Norway, Poland, Slovakia |
|  | South-Western | Portugal, Spain |
|  | Central-Western | Belgium, France, Germany, Italy, Switzerland |
| **(B) Eradication categories and therapies** | | |
|  | Triple-CA | PPI+C+A |
|  | Triple-CM | PPI+C+M |
|  | Triple-AM | PPI+A+M |
|  | Triple-AL | PPI+A+L |
|  | Conco-CAM/T | Concomitantly PPI+C+A+T, or PPI+C+A+M |
|  | Seq-CAM/T | Alternatively, PPI+C+A+T, or PPI+C+A+M |
|  | Quad- MTcB | PPI+M+Tc+Bi |
|  | Quad-Single cap | PPI+M+Tc+Bi in a three-in-one single capsule formulation |
|  | Quad-CAB | PPI+C+A+B. |
|  | Other regimens | PPI + any other antibiotic combination |

A: amoxicillin; B: bismuth salts; C: clarithromycin; Conco: concomitant; L: levofloxacin; M: metronidazole; PPI: proton pomp inhibitor; Quad: quadruple; Seq: sequential; T: tinidazole;
Tc: tetracycline

**Table S2. Use of *S. boulardii* CNCM I-745 (Sb) Across First-Line Eradication Regimens in Europe**

| **Regimen** | **Europe** | | | | | | | |
| --- | --- | --- | --- | --- | --- | --- | --- | --- |
|  | **Eastern  (N=10,029)** | | **Central-Eastern  (N=9,765)** | | **South-Western**  **(N=16,991)** | | **Central-Western**  **(N=4,067)** | |
|  | **Control (n, %)**  **(N= 8 536)** | **Sb (n, %)**  **(N=** **1493)** | **Control (n, %)**  **(N=9 409)** | **Sb (n, %)**  **(N=** **356)** | **Control (n, %)**  **(N= 16 130)** | **Sb (n, %)**  **(N=** **861)** | **Control (n, %)**  **(N= 3 470)** | **Sb (n, %)**  **(N=** **597)** |
| Triple-CA | 2063 (24.2%) | 565 (37.8%) | 5539 (58.9%) | 208 (58.4%) | 2250 (13.9%) | 35 (4.1%) | 104 (3.0%) | 167 (28.0%) |
| Triple-CM | 19 (0.22%) | 7 (0.47%) | 916 (9.7%) | 39 (10.9%) | 90 (0.56%) | 2 (0.23%) | 3 (0.09%) | 5 (0.84%) |
| Triple-AM | 59 (0.69%) | 22 (1.47%) | 73 (0.78%) | 3 (0.84%) | 43 (0.27%) | 1 (0.12%) | 2 (0.06%) | 1 (0.17%) |
| Triple-AL | 249 (2.92%) | 18 (1.21%) | 269 (2.9%) | 46 (12.9%) | 98 (0.61%) | 5 (0.58%) | 112 (3.23%) | 6 (1.01%) |
| Conco-CAM/T | 86 (1.01%) | 3 (0.20%) | 395 (4.20%) | 0 (0%) | 6036 (37.4%) | 322 (37.4%) | 401 (11.6%) | 23 (3.85%) |
| Seq-CAM/T | 143 (1.68%) | 7 (0.47%) | 89 (0.95%) | 0 (0%) | 64 (0.40%) | 0 (0%) | 2023 (58.3%) | 10 (1.67%) |
| Quad-CAB | 4035 (47.3%) | 487 (32.6%) | 934 (9.9%) | 14 (3.9%) | 1179 (7.31%) | 26 (3.02%) | -- | -- |
| Quad-MTcB | 201 (2.36%) | 45 (3.01%) | 93 (0.99%) | 0 (0%) | 33 (0.20%) | 24 (2.79%) | 9 (0.26%) | 2 (0.34%) |
| Quad-Single cap | 14 (0.16%) | 2 (0.13%) | 205 (2.18%) | 0 (0%) | 6047 (37.5%) | 417 (48.4%) | 688 (19.8%) | 252 (42.2%) |
| Other | 1667 (19.5%) | 337 (22.6%) | 896 (9.52%) | 46 (12.9%) | 290 (1.80%) | 29 (3.37%) | 128 (3.69%) | 131 (21.9%) |

A: amoxicillin; B: bismuth salts; C: clarithromycin; Conco: concomitant; L: levofloxacin; M: metronidazole; N: number of patients; PPI: proton pump inhibitor; Quad: quadruple; Seq: sequential; T: tinidazole.

Other regimens: any regimen including a PPI+ any other antibiotic combinations than those previously described.

**Table S3. Compliance by European region, country, and eradication regimen**

| **Compliance** | |  | **Control group** | | **Sb group** | | **Total** | | **p-value** |
| --- | --- | --- | --- | --- | --- | --- | --- | --- | --- |
| **All countries, all regimens** | | N (Total) | 41,919 |  | 3,245 |  | 45,164 |  |  |
|  |  | Compliant (N, %) | 40,714 | 97.1% | 3,132 | 96.5% | 43,846 | 97.1% | 0.027 |
| **By European region** | |  |  |  |  |  |  |  |  |
| 1 | Eastern | N (Total) | 8,412 |  | 1,473 |  | 9,885 |  |  |
|  |  | Compliant (N, %) | 8,239 | 97.9% | 1,454 | 98.7% | 9,693 | 98.1% | 0.027 |
| 2 | Central-Eastern | N (Total) | 8,233 |  | 335 |  | 8,568 |  |  |
|  |  | Compliant (N, %) | 7,930 | 96.3% | 309 | 92.2% | 8,239 | 96.2% | 0.000 |
| 3 | South-Western | N (Total) | 15,872 |  | 800 |  | 16,672 |  |  |
|  |  | Compliant (N, %) | 15,493 | 97.6% | 777 | 97.1% | 16,270 | 97.6% | 0.224 |
| 4 | Central-Western | N (Total) | 3,443 |  | 446 |  | 3,889 |  |  |
|  |  | Compliant (N, %) | 3,190 | 92.7% | 413 | 92.6% | 3,603 | 92.6% | 0.515 |
| **By eradication regimen** | |  |  |  |  |  |  |  |  |
|  | Triple-CA | N (Total) | 12,303 |  | 996 |  | 13,299 |  |  |
|  |  | Compliant (N, %) | 12,028 | 97.8% | 965 | 96.9% | 12,993 | 97.7% | 0.048 |
|  | Triple-CM | N (Total) | 1,620 |  | 57 |  | 1,677 |  |  |
|  |  | Compliant (N, %) | 1,578 | 94.1% | 56 | 98.2% | 1,634 | 97.4% | 0.566 |
|  | Triple-AM | N (Total) | 923 |  | 31 |  | 954 |  |  |
|  |  | Compliant (N, %) | 890 | 96.4% | 31 | 100% | 921 | 96.5% | 0.330 |
|  | Triple-AL | N (Total) | 807 |  | 86 |  | 893 |  |  |
|  |  | Compliant (N, %) | 760 | 94.2% | 80 | 93.0% | 840 | 94.1% | 0.403 |
|  | Conco- CAM/T | N (Total) | 7,242 |  | 393 |  | 7,635 |  |  |
|  |  | Compliant (N, %) | 7,039 | 97.2% | 378 | 96.2% | 7,417 | 97.1% | 0.154 |
|  | Seq-CAM/T | N (Total) | 2,299 |  | 23 |  | 2,322 |  |  |
|  |  | Compliant (N, %) | 2,118 | 92.1% | 22 | 95.7% | 2,140 | 92.2% | 0.451 |
|  | Quad-MTcB | N (Total) | 368 |  | 67 |  | 435 |  |  |
|  |  | v | 346 | 94.0% | 66 | 98.5% | 412 | 94.7% | 0.104 |
|  | Quad-Single cap | N (Total) | 6,885 |  | 625 |  | 7,510 |  |  |
|  |  | Compliant (N, %) | 6,704 | 97.4% | 596 | 95.4% | 7,300 | 97.2% | 0.004 |
|  | Quad-CAB | N (Total) | 6,090 |  | 525 |  | 6,615 |  |  |
|  |  | Compliant (N, %) | 5,977 | 98.1% | 512 | 97.5% | 6,489 | 98.1% | 0.199 |
|  | Other regimens | N (Total) | 3,382 |  | 442 |  | 3,824 |  |  |
|  |  | Compliant (N, %) | 3,274 | 96.8% | 426 | 96.4% | 3,700 | 96.8% | 0.358 |

A: amoxicillin; B: bismuth salts; C: clarithromycin; Conco: concomitant; L: levofloxacin; M: metronidazole; N: number; PPI: proton pomp inhibitor; Quad: quadruple; Sb: *Saccharomyces boulardii* CNCM I-745 (single-strain); Seq: sequential; T: tinidazole; Tc: tetracycline

Triple-CA: PPI+C+A; Triple-CM: PPI+C+M; Triple-AM: PPI+A+M; Triple-AL: PPI+A+L; Conco- CAM/T: Concomitantly PPI+C+A+T or PPI+C+A+M; Seq- CAM/T: Alternatively PPI+C+A+T or PPI+C+A+M; Quad-MTcB: PPI+M+Tc+Bi; Quad-Single cap: PPI+M+Tc+Bi in a three-in-one single capsule formulation; Quad-CAB: PPI+C+A+B. Other regimens: PPI + any other antibiotic combination.

**Table S4. Eradication Success Rates Across European regions, countries and eradication regimen**

1. **Eradication Success Rates with and without *Sb* Across Europe and by Region**

| **Success rate** | |  | **Control group** | | **Sb group** | | **Total** | | **p-value** |
| --- | --- | --- | --- | --- | --- | --- | --- | --- | --- |
| **All countries,** | | N (Total) | 38,408 |  | 2,890 |  | 41,298 |  |  |
| **all regimens** | | Success (N, %) | 34,411 | 89.6% | 2,590 | 89.6% | 37,001 | 89.6% | 0.495 |
| **By European region** | |  |  |  |  |  |  |  |  |
| 1 | Eastern | N (Total) | 6,843 |  | 1,254 |  | 8,097 |  |  |
|  |  | Success (N, %) | 6,167 | 90.1% | 1,087 | 86.7% | 7,254 | 89.6% | 0.000 |
| 2 | Central-Eastern | N (Total) | 7,157 |  | 272 |  | 7,429 |  |  |
|  |  | Success (N, %) | 6,468 | 90.4% | 263 | 96.7% | 6,731 | 90.6% | 0.000 |
| 3 | South-Western | N (Total) | 15,591 |  | 770 |  | 16,361 |  |  |
|  |  | Success (N, %) | 13,926 | 89.3% | 711 | 92.3% | 14,637 | 89.5% | 0.005 |
| 4 | Central-Western | N (Total) | 3,124 |  | 417 |  | 3,541 |  |  |
|  |  | Success (N, %) | 2,855 | 91.4% | 372 | 89.2% | 3,227 | 91.1% | 0.086 |

A: amoxicillin; B: bismuth salts; C: clarithromycin; Conco: concomitant; L: levofloxacin; M: metronidazole; N: number; PPI: proton pomp inhibitor; Quad: quadruple; Sb: *Saccharomyces boulardii* CNCM I-745 (single-strain); Seq: sequential; T: tinidazole; Tc: tetracycline

Triple-CA: PPI+C+A; Triple-CM: PPI+C+M; Triple-AM: PPI+A+M; Triple-AL: PPI+A+L; Conco- CAM/T: Concomitantly PPI+C+A+T or PPI+C+A+M; Seq- CAM/T: Alternatively PPI+C+A+T or PPI+C+A+M; Quad-MTcB: PPI+M+Tc+Bi; Quad-Single cap: PPI+M+Tc+Bi in a three-in-one single capsule formulation; Quad-CAB: PPI+C+A+B. Other regimens: PPI + any other antibiotic combination.

1. **Eradication Success Rates with and without *Sb* in main countries and by eradication regimen**

| **Success rate** | |  | **Control group** | | **Sb group** | | **Total** | | **p-value** |
| --- | --- | --- | --- | --- | --- | --- | --- | --- | --- |
| Italy | Triple-CA | N (Total) | 46 |  | 131 |  | 177 |  |  |
|  |  | Success (N, %) | 36 | 78.3% | 108 | 82.4% | 131 | 81.4% | 0.336 |
|  | Triple-AL | N (Total) | 101 |  | 6 |  | 107 |  |  |
|  |  | Success (N, %) | 80 | 79.2% | 6 | 100.0% | 86 | 80.4% | 0.500 |
|  | Conco- CAM/T | N (Total) | 384 |  | 18 |  | 402 |  |  |
|  |  | Success (N, %) | 367 | 95.6% | 15 | 83.3% | 382 | 95.0% | 0.053 |
|  | Seq- CAM/T | N (Total) | 1,789 |  | 6 |  | 1,795 |  |  |
|  |  | Success (N, %) | 1,620 | 90.6% | 6 | 100.0% | 1,626 | 90.6% | 0.552 |
|  | Quad-Single cap | N (Total) | 520 |  | 210 |  | 730 |  |  |
|  |  | Success (N, %) | 500 | 96.2% | 198 | 94.3% | 698 | 95.6% | 0.179 |
|  | Total | N (Total) | 2,954 |  | 400 |  | 3,354 |  |  |
|  |  | Success (N, %) | 2,691 | 91.1% | 355 | 88.8% | 3,046 | 90.8% | 0.078 |
| Russia | Triple-CA | N (Total) | 438 |  | 29 |  | 467 |  |  |
|  |  | Success (N, %) | 368 | 84.0% | 19 | 65.5% | 387 | 82.9% | 0.015 |
|  | Quad-CAB | N (Total) | 1,788 |  | 71 |  | 1,859 |  |  |
|  |  | Success (N, %) | 1,691 | 94.6% | 66 | 93.0% | 1,757 | 94.5% | 0.349 |
|  | Total | N (Total) | 2,917 |  | 123 |  | 3,040 |  |  |
|  |  | Success (N, %) | 2,679 | 91.8% | 102 | 82.9% | 2,781 | 91.5% | 0.001 |
| Spain | Triple-CA | N (Total) | 2,064 |  | 31 |  | 2,095 |  |  |
|  |  | Success (N, %) | 1,681 | 81.4% | 25 | 80.6% | 1,706 | 81.4% | 0.528 |
|  | Conco- CAM/T | N (Total) | 5,674 |  | 285 |  | 5,959 |  |  |
|  |  | Success (N, %) | 5,075 | 89.4% | 270 | 94.7% | 5,345 | 89.7% | 0.001 |
|  | Quad-Single cap | N (Total) | 5,653 |  | 375 |  | 6,028 |  |  |
|  |  | Success (N, %) | 5,273 | 93.3% | 343 | 91.5% | 5,616 | 93.2% | 0.110 |
|  | Quad-CAB | N (Total) | 1,147 |  | 24 |  | 1,171 |  |  |
|  |  | Success (N, %) | 1,046 | 91.2% | 20 | 83.3% | 1,066 | 91.0% | 0.161 |
|  | Total | N (Total) | 15,069 |  | 766 |  | 15,835 |  |  |
|  |  | Success (N, %) | 13,467 | 89.4% | 707 | 92.3% | 14,174 | 89.5% | 0.006 |
| Czech | Triple-CA | N (Total) | 175 |  | 42 |  | 217 |  |  |
| Republic |  | Success (N, %) | 147 | 84.0% | 41 | 97.6% | 188 | 86.6% | 0.011 |
|  | Total | N (Total) | 331 |  | 54 |  | 385 |  |  |
|  |  | Success (N, %) | 283 | 85.5% | 81 | 94.4% | 334 | 86.8% | 0.048 |
| Azerbaijan | Triple-CA | N (Total) | 2,158 |  | 92 |  | 2,250 |  |  |
|  |  | Success (N, %) | 1,974 | 91.5% | 89 | 96.7% | 2,063 | 91.7% | 0.044 |
|  | Triple-CM | N (Total) | 657 |  | 29 |  | 686 |  |  |
|  |  | Success (N, %) | 615 | 93.6% | 29 | 100.0% | 644 | 93.9% | 0.154 |
|  | Triple-AL | N (Total) | 176 |  | 42 |  | 218 |  |  |
|  |  | Success (N, %) | 161 | 91.5% | 40 | 95.2% | 201 | 92.2% | 0.327 |
|  | Quad-CAB | N (Total) | 684 |  | 12 |  | 696 |  |  |
|  |  | Success (N, %) | 677 | 99.0% | 12 | 100.0% | 689 | 99.0% | 0.885 |
|  | Total | N (Total) | 3,886 |  | 207 |  | 4,093 |  |  |
|  |  | Success (N, %) | 3,627 | 93.3% | 201 | 97.1% | 3,828 | 93.5% | 0.016 |

A: amoxicillin; B: bismuth salts; C: clarithromycin; Conco: concomitant; L: levofloxacin; M: metronidazole; N: number; PPI: proton pomp inhibitor; Quad: quadruple; Sb: *Saccharomyces boulardii* CNCM I-745 (single-strain); Seq: sequential; T: tinidazole; Tc: tetracycline

Triple-CA: PPI+C+A; Triple-CM: PPI+C+M; Triple-AM: PPI+A+M; Triple-AL: PPI+A+L; Conco- CAM/T: Concomitantly PPI+C+A+T or PPI+C+A+M; Seq- CAM/T: Alternatively PPI+C+A+T or PPI+C+A+M; Quad-MTcB: PPI+M+Tc+Bi; Quad-Single cap: PPI+M+Tc+Bi in a three-in-one single capsule formulation; Quad-CAB: PPI+C+A+B.

**Table S5. Multivariate analysis of factors associated with eradication effectiveness across H. pylori treatment regimens**

1. **Multivariate Logistic Regression Identifying Factors Associated with the Effectiveness of Bismuth Quadruple Therapy**

| **Variable** | **Category** | **OR** | **Lower CI** | **Upper CI** | **p-value** |
| --- | --- | --- | --- | --- | --- |
| Compliance | <90% vs ≥90% | 4.677 | 1.840 | 11.891 | 0.001 |
| PPI potency | Low dose | Reference |  |  |  |
|  | Standard dose | 1.176 | 0.689 | 2.008 | 0.551 |
|  | High dose | 3.575 | 1.563 | 8.176 | 0.003 |
| Rebamipide use | Yes vs No | 5.705 | 2.093 | 15.552 | 0.001 |

1. **Multivariate Logistic Regression Identifying Factors Associated with the Effectiveness of Standard Triple Therapy**

| Variable | Category | OR | Lower CI | Upper CI | p-value |
| --- | --- | --- | --- | --- | --- |
| Gender | Male vs Female | 1.269 | 1.129 | 1.427 | 0.000 |
| Compliance | <90% vs ≥90% | 18.666 | 13.734 | 25.369 | 0.000 |
| Indication | Ulcer vs Non-ulcer | 1.271 | 1.096 | 1.474 | 0.002 |
| PPI potency | Low dose | Reference |  |  |  |
|  | Standard dose | 1.439 | 1.265 | 1.638 | 0.000 |
|  | High dose | 1.916 | 1.599 | 2.296 | 0.000 |
| Treatment duration | 7 days | Reference |  |  |  |
|  | 10 days | 1.459 | 1.241 | 1.716 | 0.000 |
|  | 14 days | 2.055 | 1.730 | 2.440 | 0.000 |
| Region | Central-Western | Reference |  |  |  |
|  | East | 1.962 | 1.678 | 2.294 | 0.000 |
|  | Central-Eastern | 1.054 | 0.909 | 1.223 | 0.486 |
|  | South-Western | 2.077 | 1.507 | 2.861 | 0.000 |

1. **Multivariate Logistic Regression Identifying Factors Associated with the Effectiveness of Single-Capsule Bismuth Quadruple Therapy**

| Variable | Category | OR | Lower CI | Upper CI | p-value |
| --- | --- | --- | --- | --- | --- |
| Compliance | <90% vs ≥90% | 14.004 | 9.654 | 20.314 | 0.000 |
| PPI potency |  | Reference |  |  |  |
|  | Standard dose | 1.777 | 1.392 | 2.267 | 0.000 |
|  | High dose | 1.699 | 1.354 | 2.132 | 0.000 |
| Region | Central-Western | Reference |  |  |  |
|  | East | 1.371 | 0.152 | 12.401 | 0.779 |
|  | Central-Eastern | 3.084 | 0.356 | 26.750 | 0.307 |
|  | South-Western | 5.571 | 0.627 | 49.492 | 0.123 |

**Table S6. Overall safety in the Sb and control groups**

| **Adverse events** | |  | **Control group** | | **Sb group** | | **Total** | | **p-value** |
| --- | --- | --- | --- | --- | --- | --- | --- | --- | --- |
|  | Any* | N (Total) | 41,168 |  | 3,284 |  | 44,452 |  |  |
|  |  | With at least one (N, %) | 9,054 | 22.0% | 700 | 21.3% | 9,754 | 21.9% | 0.189 |
| **By adverse event** | | |  |  |  |  |  |  |  |
|  | Dysgeusia | N (Total) | 44219 |  | 3,541 |  | 47,760 |  |  |
|  |  | With at least one (N, %) | 3,049 | 6.9% | 187 | 5.3% | 3,236 | 6.8% | 0.000 |
|  | Diarrhea | N (Total) | 44,219 |  | 3,541 |  | 47,760 |  |  |
|  |  | With at least one (N, %) | 3,300 | 7.5% | 184 | 5.2% | 3,484 | 7.3% | 0.000 |
|  | Nausea | N (Total) | 44,219 |  | 3,541 |  | 47,760 |  |  |
|  |  | With at least one (N, %) | 3,246 | 7.3% | 155 | 4.4% | 3,401 | 7.1% | 0.000 |
|  | Vomiting | N (Total) | 44,219 |  | 3,541 |  | 47,760 |  |  |
|  |  | With at least one (N, %) | 1,225 | 2.8% | 89 | 2.5% | 1,314 | 2.8% | 0.199 |
|  | Dyspepsia | N (Total) | 44,219 |  | 3,541 |  | 47,760 |  |  |
|  |  | With at least one (N, %) | 1,475 | 3.3% | 89 | 2.5% | 1,564 | 3.3% | 0.005 |
|  | Heartburn | N (Total) | 44,219 |  | 3,541 |  | 47,760 |  |  |
|  |  | With at least one (N, %) | 803 | 1.8% | 38 | 1.1% | 841 | 1.8% | 0.001 |
|  | Abdominal pain | N (Total) | 44,219 |  | 3,541 |  | 47,760 |  |  |
|  |  | With at least one (N, %) | 1,676 | 3.8% | 79 | 2.2% | 1,755 | 3.7% | 0.000 |
|  | Asthenia | N (Total) | 44,219 |  | 3,541 |  | 47,760 |  |  |
|  |  | With at least one (N, %) | 1,778 | 4.0% | 59 | 1.7% | 1,837 | 3.8% | 0.000 |
|  | Anorexia | N (Total) | 44,219 |  | 3,541 |  | 47,760 |  |  |
|  |  | With at least one (N, %) | 1,316 | 3.0% | 10 | 0.3% | 1,326 | 2.8% | 0.000 |
| **By European region** | | |  |  |  |  |  |  |  |
| 1 | Eastern | N (Total) | 8,509 |  | 1,476 |  | 9,985 |  |  |
|  |  | With at least one (N, %) | 2,242 | 26.3% | 406 | 27.5% | 2,648 | 26.5% | 0.184 |
| 2 | Central- | N (Total) | 7,338 |  | 348 |  | 7,686 |  |  |
|  | Eastern | With at least one (N, %) | 1,172 | 16.0% | 28 | 8.0% | 1,200 | 15.6% | 0.000 |
| 3 | South- | N (Total) | 15,856 |  | 809 |  | 16,665 |  |  |
|  | Western | With at least one (N, %) | 4,121 | 26.0% | 94 | 11.6% | 4,215 | 25.3% | 0.000 |
| 4 | Central- | N (Total) | 3,438 |  | 452 |  | 3,890 |  |  |
|  | Western | With at least one (N, %) | 686 | 20.0% | 143 | 31.6% | 829 | 21.3% | 0.000 |
| **By country** | |  |  |  |  |  |  |  |  |
| 1 | Albania | N (Total) | 29 |  | 17 |  | 46 |  |  |
|  |  | With at least one (N, %) | 1 | 3.4% | 4 | 23.5% | 5 | 10.9% | 0.055 |
|  | Bulgaria | N (Total) | 197 |  | - |  | 197 |  |  |
|  |  | With at least one (N, %) | 8 | 4.1% | - | - | 8 | 4.1% | - |
|  | Romania | N (Total) | 135 |  | - |  | 135 |  |  |
|  |  | With at least one (N, %) | 39 | 28.9% | - | - | 39 | 28.9% | - |
|  | Russia | N (Total) | 7,092 |  | 1,415 |  | 8,507 |  |  |
|  |  | With at least one (N, %) | 2,119 | 29.9% | 401 | 28.3% | 2,520 | 29.6% | 0.130 |
|  | Turkey | N (Total) | 279 |  | 19 |  | 298 |  |  |
|  |  | With at least one (N, %) | 69 | 24.7% | 1 | 5.3% | 70 | 23.5% | 0.038 |
|  | Ukraine | N (Total) | 777 |  | 25 |  | 802 |  |  |
|  |  | With at least one (N, %) | 6 | 0.8% | 0 | 0.0% | 6 | 0.7% | 0.826 |
| 2 | Azerbaijan | N (Total) | 4,219 |  | 224 |  | 4,443 |  |  |
|  |  | With at least one (N, %) | 478 | 11.3% | 28 | 12.5% | 506 | 11.4% | 0.327 |
|  | Czech Republic | N (Total) | 337 |  | 84 |  | 421 |  |  |
|  |  | With at least one (N, %) | 10 | 3.0% | 0 | 0.0% | 10 | 2.4% | 0.105 |
|  | Greece | N (Total) | 495 |  | - |  | 495 |  |  |
|  |  | With at least one (N, %) | 170 | 34.3% | - | - | 170 | 34.3% | - |
|  | Hungary | N (Total) | 298 |  | - |  | 298 |  |  |
|  |  | With at least one (N, %) | 104 | 34.9% | - | - | 104 | 34.9% | - |
|  | Latvia | N (Total) | 525 |  | - |  | 525 |  |  |
|  |  | With at least one (N, %) | 255 | 48.6% | - | - | 255 | 48.6% | - |
|  | Lithuania | N (Total) | 1,079 |  | - |  | 1,079 |  |  |
|  |  | With at least one (N, %) | 101 | 9.4% | - | - | 101 | 9.4% | - |
|  | Malta | N (Total) | - |  | 35 |  | 35 |  |  |
|  |  | With at least one (N, %) | - | - | 0 | - | 0 | - | - |
|  | Norway | N (Total) | 646 |  | - |  | 646 |  |  |
|  |  | With at least one (N, %) | 379 | 58.7% | - | - | 379 | 58.7% | - |
|  | Poland | N (Total) | 383 |  | - |  | 383 |  |  |
|  |  | With at least one (N, %) | 53 | 13.8% | - | - | 53 | 13.8% | - |
|  | Slovakia | N (Total) | 2 |  | 5 |  | 7 |  |  |
|  |  | With at least one (N, %) | 1 | 50.0% | 0 | 0.0% | 1 | 14.3% | 0.286 |
| 3 | Portugal | N (Total) | 530 |  | 5 |  | 535 |  |  |
|  |  | With at least one (N, %) | 60 | 11.3% | 0 | 0.0% | 60 | 11.2% |  |
|  | Spain | N (Total) | 15,326 |  | 804 |  | 16,130 |  |  |
|  |  | With at least one (N, %) | 4,061 | 26.5% | 94 | 11.7% | 4,155 | 25.8% | 0.000 |
| 4 | Belgium | N (Total) | 25 |  | 4 |  | 29 |  |  |
|  |  | With at least one (N, %) | - | - | - | - | - | - | - |
|  | France | N (Total) | 4 |  | 13 |  | 17 |  |  |
|  |  | With at least one (N, %) | 0 | 0.0% | 6 | 46.2% | 6 | 35.3% | 0.139 |
|  | Germany | N (Total) | 155 |  | - |  | 155 |  |  |
|  |  | With at least one (N, %) | 5 | 3.2% | - | - | 5 | 3.2% | - |
|  | Italy | N (Total) | 3,254 |  | 435 |  | 3,689 |  |  |
|  |  | With at least one (N, %) | 681 | 20.9% | 137 | 31.5% | 818 | 22.2% | 0.000 |
|  | Switzerland | N (Total) | 127 |  | - |  | 127 |  |  |
|  |  | With at least one (N, %) | 9 | 7.1% | - | - | 9 | 7.1% | - |

*Meaning the incidence of at least one adverse event. A: amoxicillin; B: bismuth salts; C: clarithromycin; L: levofloxacin; M: metronidazole; N: number; PPI: proton pomp inhibitor; Sb: *Saccharomyces boulardii* CNCM I-745 (single-strain); T: tinidazole; Tc: tetracycline.

**Table S7. Safety of each treatment by European region**

| **European region** | **Eradication therapy** | | **Control group** | | **Sb group** | | **Total** | | **p-value** |
| --- | --- | --- | --- | --- | --- | --- | --- | --- | --- |
| Eastern | N |  | 2,298 |  | 354 |  | 2,652 |  | 0.006 |
|  | Triple CA: at least one AE | N, % | 534 | 23.2% | 105 | 29.7% | 639 | 24.1% |  |
|  | Quad-CAB | N, % | 1,764 | 76.8% | 249 | 70.4% | 2,013 | 75.9% |  |
| Central-Eastern | N |  | 1,018 |  | 48 |  | 1,066 |  | 0.000 |
|  | Triple-CA | N, % | 623 | 61.2% | 12 | 25.0% | 635 | 59.6% |  |
|  | Triple-AL | N, % | 395 | 38.8% | 36 | 75.0% | 431 | 40.4% |  |
| South-Western | N |  | 4,153 |  | 256 |  | 4,409 |  | 0.000 |
|  | Quad-Single cap | N, % | 2,413 | 58.1% | 218 | 85.2% | 2,631 | 59.7% |  |
|  | Quad-CAM | N, % | 1,740 | 41.9% | 38 | 14.8% | 1,778 | 40.3% |  |
| Central-Western | N |  | 2,431 |  | 254 |  | 2,685 |  | 0.000 |
|  | Triple-CA | N, % | 18 | 0.7% | 36 | 14.2% | 54 | 2.0% |  |
|  | Quad-Single cap | N, % | 2,413 | 99.3% | 218 | 85.8% | 2,631 | 98.0% |  |

A: amoxicillin; B: bismuth salts; C: clarithromycin; L: levofloxacin; M: metronidazole; N, %: number and percentage of treatments with at least one adverse event; Quad: quadruple; Sb: *Saccharomyces boulardii* CNCM I-745 (single-strain)

Triple-CA: PPI+C+A; Triple-AL: PPI+A+L; Quad-Single cap: PPI+M+Tc+Bi in a three-in-one single capsule formulation; Quad-CAB: PPI+C+A+Bi; Quad-CAM: PPI+C+A+M.

Percentages are calculated within the treatment group (with N as denominator).
